# Quantifying the Landscape and Transition Paths for Proliferation–Quiescence Fate Decisions

**DOI:** 10.3390/jcm9082582

**Published:** 2020-08-10

**Authors:** Zihao Chen, Chunhe Li

**Affiliations:** 1 Shanghai Center for Mathematical Sciences, Fudan University, Shanghai 200433, China; 19210850009@fudan.edu.cn; 2 Institute of Science and Technology for Brain-Inspired Intelligence, Fudan University, Shanghai 200433, China

**Keywords:** cell cycle, landscape, proliferation–quiescence decision, kinetic paths, checkpoints

## Abstract

The cell cycle, essential for biological functions, experiences delicate spatiotemporal regulation. The transition between G1 and S phase, which is called the proliferation–quiescence decision, is critical to the cell cycle. However, the stability and underlying stochastic dynamical mechanisms of the proliferation–quiescence decision have not been fully understood. To quantify the process of the proliferation–quiescence decision, we constructed its underlying landscape based on the relevant gene regulatory network. We identified three attractors on the landscape corresponding to the G0, G1, and S phases, individually, which are supported by single-cell data. By calculating the transition path, which quantifies the potential barrier, we built expression profiles in temporal order for key regulators in different transitions. We propose that the two saddle points on the landscape characterize restriction point (RP) and G1/S checkpoint, respectively, which provides quantitative and physical explanations for the mechanisms of Rb governing the RP while p21 controlling the G1/S checkpoint. We found that Emi1 inhibits the transition from G0 to G1, while Emi1 in a suitable range facilitates the transition from G1 to S. These results are partially consistent with previous studies, which also suggested new roles of Emi1 in the cell cycle. By global sensitivity analysis, we identified some critical regulatory factors influencing the proliferation–quiescence decision. Our work provides a global view of the stochasticity and dynamics in the proliferation–quiescence decision of the cell cycle.

## 1. Introduction

Mammalian cells undergo strictly ordered G1->S->G2->M phases driven by cyclins and cyclin-dependent kinases (Cdks) [1]. When cells suffer mild DNA damage or nutritional deficiencies, they can enter a reversible state of growth arrest known as G0 quiescent state, which is important to preventing the propagation of mutations. The cell cycle escapes from the quiescent state at the same point after restoring nutrition [2]. This unique checkpoint is called the restriction point (RP) [3]. The RP and another checkpoint, the G1/S checkpoint, play critical roles in the proliferation–quiescence decision, and underlying regulatory networks have been proposed [4,5,6,7,8,9,10,11]. Previous studies have explored the proliferation–quiescence cell fate decision using mathematical modeling and experimental approaches [12]. However, the global stability of the proliferation–quiescence decision network, especially the dynamical mechanisms for the proliferation–quiescence fate transitions, remain to be clarified.

Cyclins–Cdks hyperphosphorylate the hypophosphorylated retinoblastoma protein (pRb) to cease the quiescent state and initiate the cell cycle [13]. CycD–Cdk4/6 (CycD) establishes the prereplication complex (pRC) [14] while CycE–Cdk2 (CycE) and CycA–Cdk2 (CycA) drive the G1 to S transition [15,16]. A series of Cdk inhibitors (CKIs) have been identified to inhibit the cyclins–Cdks [13]. One of CKIs, p21, is vital to the p53-mediated response to DNA damage [17,18]. E2F inhibited by pRb [19] serves as an essential switch under the RP [20]. E2F is promoted by CycA and CycE, which forms positive feedback loops [21]. Multiple E3 ligases mediate E2F degradation, the anaphase-promoting complex/cyclosome (APC/C). The active form of APC/C, $APC/C^{Cdh1}$ (Cdh1), is inhibited by the early mitotic inhibitor (Emi1) [22]. Cdh1 targets CycA and E2F during the G1 phase [20].

Recently, Heldt et al. proposed that the proliferation–quiescence decision can be understood as two bistable switches based on a gene network model [12]. However, it remains challenging to quantify the stochastic transitional dynamics of this process. The Waddington epigenetic landscape, as a metaphor, has been used to describe the cell fate decision processes [23]. The energy landscape theory has also been developed to the study protein structures and dynamics of cellular networks [24,25,26,27,28,29]. In this work, based on the landscape theory, we quantified the potential energy landscape of proliferation–quiescence fate decision based on underlying gene regulatory networks of the cell cycle. We identified three attractors on the landscape, which characterize G0, G1, and S states of the cell cycle, individually. We propose that the proliferation–quiescence decision in early mitosis can be understood as a tristable switch, which agrees well with single-cell data [30]. The tristable landscape provides a global view of proliferation–quiescence fate decision, and quantitative measures for the feasibility of transitions among G0, G1, and S phases in the cell cycle. To quantify the effects of key regulators, we calculated the transition actions by characterizing the potential barriers to measure the feasibility of the transitions. The expression profiles of regulators in temporal order demonstrate that different genes are switched on or off in different orders for both G0 to G1 and G1 to S transitions.

We need to stress that a major point of this work is to quantify the global stability for the proliferation–quiescence decision process based on energy landscape theory, which differs from the traditional modeling approach only based on ordinary differential equations (ODEs) [12]. The “global stability” is defined as compared to “local stability” in ODEs and bifurcation theories [25,26,27,28,29]. In bifurcation theory (local stability analysis), the equilibrium points (static solutions of ODEs) can be classified to stable or unstable points. However, local stability analysis cannot tell which is more stable for the two stable equilibrium points. In biological systems, this is important because if one stable point is much less stable than another stable point, it may never being observed due to the fluctuations in biological systems. Thus, the “global stability”, or relative stability, is used to address this problem. In other words, the “global stability” tells us which stable point is more stable considering the stochasticity. From a energy landscape view, if a basin is deeper, that means this basin (cell type) is more stable, globally.

The “energy landscape theory” provides a powerful tool with which to study “global stability” in cellular systems. As a metaphor, the Waddington epigenetic landscape has been used to describe the cell fate decision processes [21]. From the perspective of the energy landscape, different cell types are described as attractors (or basins) on a potential surface. The cell differentiation process is viewed as a ball rolling from one basin to the other on the landscape surface by going over certain energy barriers. More importantly, the barrier heights among the attractors provide a measure with which to quantify the feasibility of the cell transformation from one cell type (basin) to the other. For a long time, the epigenetic landscape has been suggested by Waddington only as a metaphor with which to understand cell fate decision process. Recent works showed that we can quantify the energy landscape with mathematical modeling [22,23,24,25,26,27,31,32,33,34]. In this work, we aimed to use the energy landscape theory to study the stochastic transition dynamics of the proliferation–quiescence decision.

## 2. Materials and Methods

### 2.1. Mathematical Modeling

**Figure 1:**
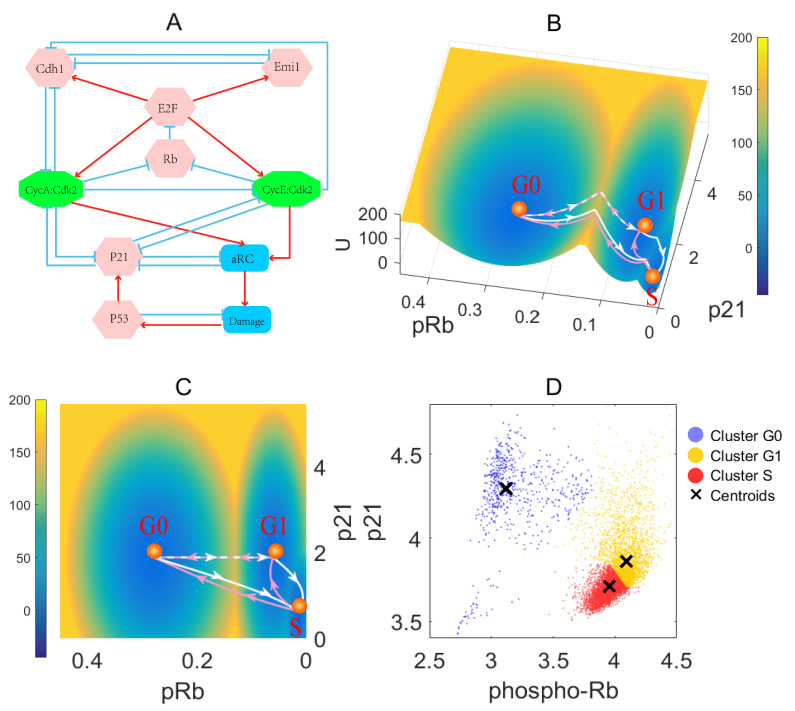
(**A**) The influence diagram of an early cell-cycle model. The red arrows represent activation and the blue bars represent inhibition. Pink hexagons represent transcription factors, green octagons represent protein complexes, and blue quadrilaterals represent cellular components of non-simple structures, including cell damage and cell replication-activated complexes. (**B**) The landscape of the proliferation–quiescence fate decision and transition path in 3D view. The white arrows represent the direction in which the cell cycle proceeds, and the pink arrows represents the backward direction of the cell cycle. The pRb axis represents the relative concentration of pRb, and the p21 axis represents the relative concentration of p21 (unit: AU). The U axis represents the potential energy. The G0 state represents the cell cycle cannot pass the RP and enters the G0 phase. The G1 state represents that the cell cannot pass the G1/S checkpoint and thus halt in G1. The S state represents that the cell cycle proceeds to the replication stage. The transition path represents the most probable path for transitions by minimizing the transition action (Equation (8)). (**C**) The landscape and transition path in 2D view. (**D**) The single-cell data for the phosphorylation level of Rb and the concentration of p21. The phospho-Rb axis denotes the phosphorylation level of Rb, including the hypo and hyperphosphorylated Rb. The p21 axis denotes the p21 concentration. Each point corresponds to a single cell.

The proliferation–quiescence decision is controlled by a series of regulatory pathways. The positive feedback loops between E2F and cyclins release cell cycle from the quiescence caused by Rb [35]. Cdh1 and p21 inhibit the cyclins and prevent the initialization of the active replication complex (aRC) [36]. The DNA damage occurring randomly leads to the G0 quiescence and G1 arrest. Activation of aRC increases the risk of DNA damage and forms a negative feedback loop through the p53-mediated pathway [12]. These signaling events ensure the robustness of cell cycle control and compose the underlying regulatory network (Figure 1A) [12,35].

Recently, Heldt et al. constructed an ordinary differential equations (ODEs) model to describe proliferation–quiescence decision [12]. To focus on the key molecular events for the decision-making of proliferation, we made some modifications to this model and obtained our ODEs model. The model involves some important proteins related to the cell cycle, such as E2F, cyclin E–Cdk2, cyclin A–Cdk2, and Emi1 and their interactions to describe the underlying molecular mechanism of RP and the G1/S checkpoint. The ODE models and parameters are provided in Appendix A and Appendix B).

**Figure 2:**
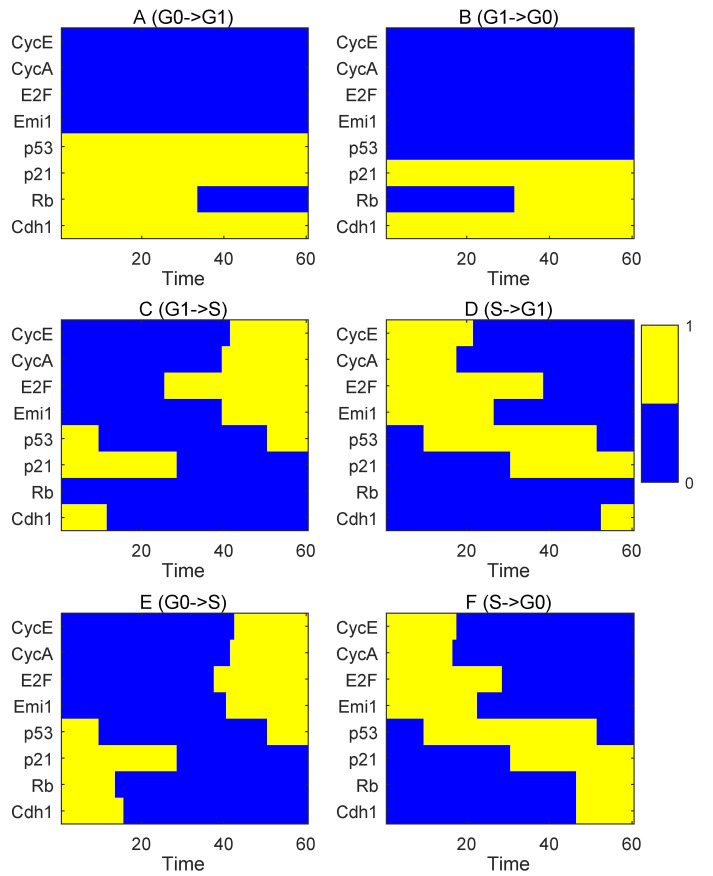
The kinetic transition path for each state transition. (**A**) The transition path from G0 to G1. (**B**) The transition path from G1 to G0. (**C**) The transition path from G1 to S. (**D**) The transition path from S to G1. (**E**) The transition path from G0 to S. (**F**) The transition path from S to G0. The blue bars denote inactivated states, and the yellow bars denote activated states. The X axis represents the time of cell state transition and the Y axis represent different regulators.

**Figure 3:**
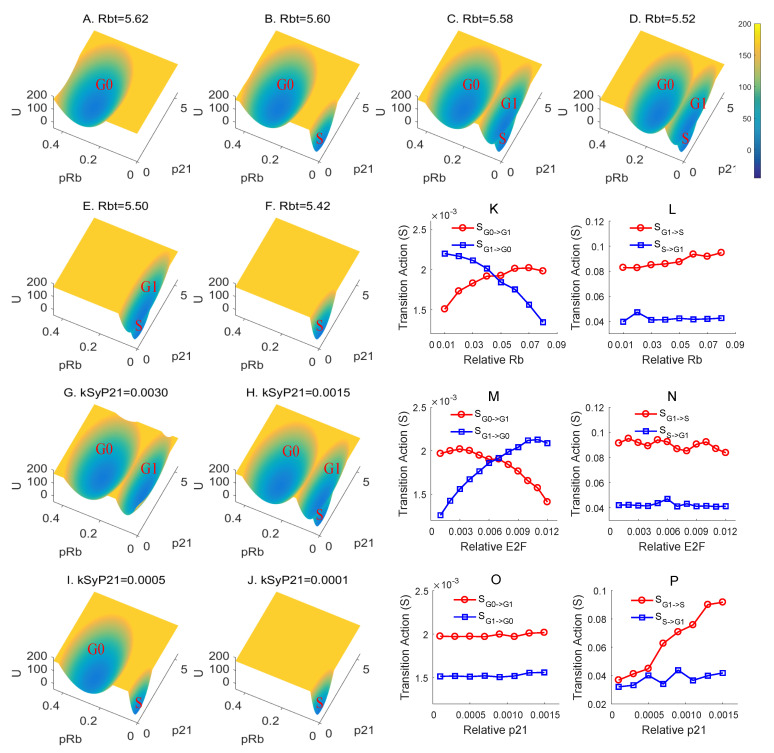
(**A**–**F**) Landscape changes as the Rb concentrations change. Rbt is the total amount of Rb, including various isoforms. From (**A**) to (**F**), the total concentration of Rb decreases. The pRb axis represents the relative concentration of Rb. The p21 axis represents the relative concentration of p21. The U axis is the potential energy. (**G**–**J**) Landscape changes as the synthesis rates of p21 (kSyp21) changes. From G to J, the synthesis rate of p21 decreases. The pRb axis represents the relative concentration of Rb. The p21 axis represents the relative concentration of p21. The U axis is the potential energy. (**K**–**P**) Effect of Rb (**K**,**L**), E2F (**M**,**N**), and p21 (**O**,**P**) changes on the transition action (S) between G0-G1 and G1-S. (**K**) The effect of Rb on the transition action value between G0/G1. The x axis represents the relative concentration of Rb, and the y axis represents the value of the minimum action. The red line represents the amount of action that is required to convert G0 to G1 and the blue line represents the amount of action that required to convert G1 to G0. (**L**) The effect of Rb on the transition action (S) between G1-S. The same for E2F (M-N) and p21 (**O**,**P**).

**Figure 4:**
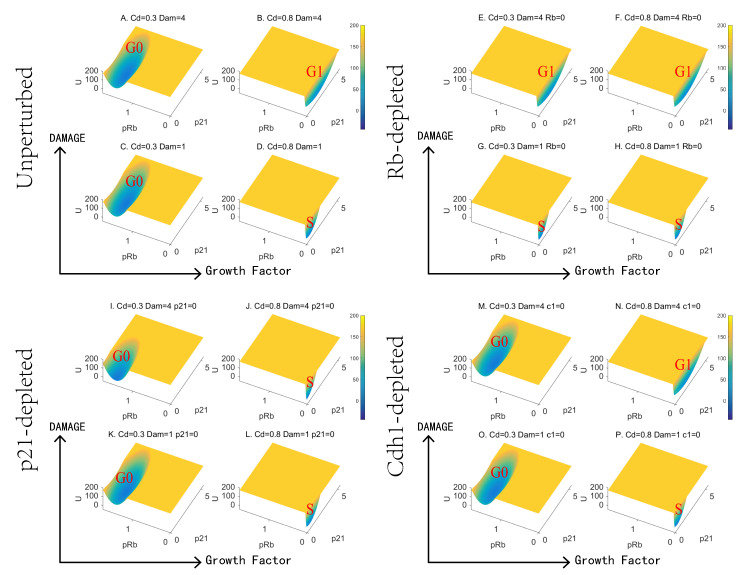
Landscape changes as growth factor and DNA damage change at different conditions. (**A**–**D**) Landscape of the proliferation–quiescence decision for an unperturbed system. (**E**–**H**) Landscape with Rb depletion. (**I**–**L**) Landscape with p21 depletion. (**M**–**P**) Landscape with Cdh1 depletion. The DNA damage level increases from bottom to top and the growth factor induced by CycD increases from left to right.

**Figure 5:**
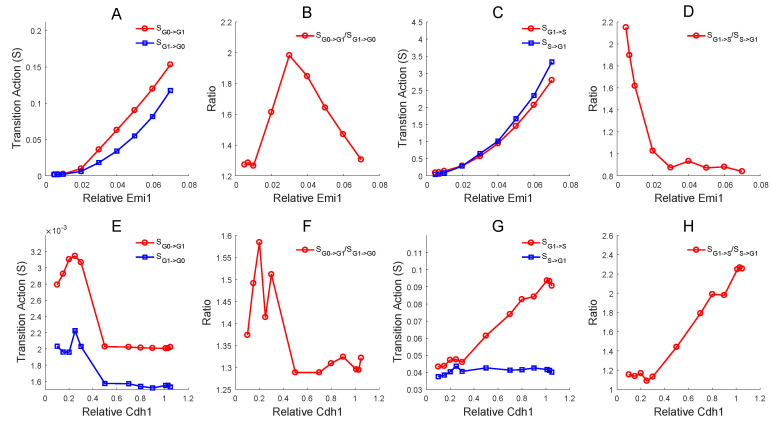
Effects of Emi1 (**A**–**D**) and Cdh1 (**E**–**H**) on the transition action (S) between G0 and G1 and between G1 and S. (**A**) The effect of Emi1 on the transition action between G0 and G1. The x axis represents the relative concentration of Emi1, and the y axis represents the value of the transition action (S). The red line represents the action from G0 to G1 and the blue line represents the action from G1 to G0. (**B**) The ratio of the action between G0->G1 and G1->G0 changes with Emi1. (**C**) The effect of Emi1 on the transition action (S) between G1 and S. (**D**) The ratio of the action between G1->S and S->G1 changes with Emi1. (**E**) The effect of Cdh1 on the transition action (S) between G0 and G1. (**F**) The ratio of the action between G0->G1 and G1->G0 changes with Cdh1. (**G**) The effect of Cdh1 on the transition action (S) between G1 and S. (**H**) The ratio of the action between G1->S and S->G1 changes with Cdh1.

**Figure 6:**
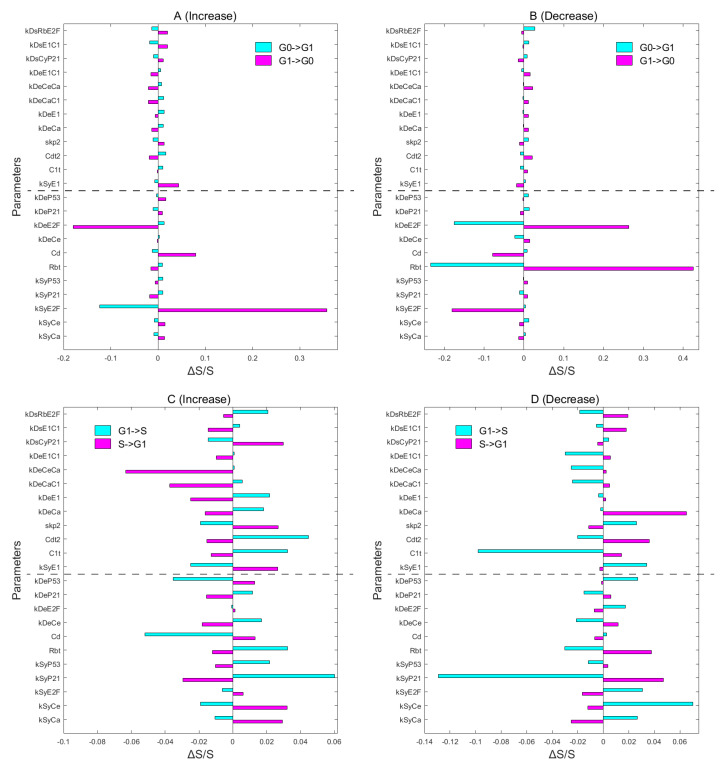
Sensitivity analysis for the 23 parameters on the transition action of G0->G1 (**A**), G1->G0 (**B**), G1->S (**C**), and S->G1 (**D**). The x axis represents the relative change of transition action in terms of unperturbed system, denoted by ΔS/S. Some sensitive parameters were increased or reduced by 0.5% (below the dotted line), including kSyCe, kSyCa, kSyE2F, kSyP21, kSyP53, Rbt, Cd, kDeCe, kDeE2F, kDeP21, abd kDeP53, while the other parameters were increased or decreased by 5% (above the dotted line). (**A**,**B**) the magenta bar represents the G1->G0 transition, and the cyan bar represents the G0->G1 transition. (**A**) Each parameter was increased for the transition between G0 and G1. (**B**) Each parameter was decreased for the transition between G0 and G1. (**C**,**D**) The magenta bar represents the S->G1 transition, and the cyan bar represents the G1->S transition. (**C**) Each parameter was increased for the transition between G1 and S. (**D**) Each parameter was decreased for the transition between G1 and S.

We provide the description of how to estimate the model parameters (see the first section in Supporting Information). In fact, this is very challenging because of the huge state space of the system. For such a high-dimensional nonlinear ODEs system, very few analytical techniques can be used to perform this job. Thus, our way is that, for each parameter set, we solve ODEs numerically by giving random initial conditions (1000 random initial conditions) to the 20-dimensional system, and calculate the results in steady state. There is evidence showing that G1 and S are not one single state [12,30]. Gookin et al. obtained three clusters for single-cell data (see Figures S3 and S4) [30]. These results based on single-cell data strongly support there are three clusters (three stable cell states) in cell cycle decisions. Thus, one major criterion for parameter estimations is whether the system can display three stable cell states that can match G0, G1, and S states. After carefully tuning the parameters, we finally obtained the tristable state. In minimum action section and potential barrier section, and in global sensitivity analysis section, we did the perturbation for all the parameters, which showed the relative robustness of current parameter regions. We provide the results of different parameters for different genes in Figure 2, Figure 3, Figure 4, Figure 5 and Figure 6 and Figures S6–S19.

### 2.2. Self Consistent Mean Field Approximation

As we know, the Waddington landscape is only a metaphor. Here, we aim to quantify the landscape with mathematical models. One way of doing that is to obtain the high-dimensional probability distribution based on the ODE models (Appendix A). According to U=−log(Pss), we can get the energy landscape *U* [25,26,27,28]. Here, Pss is steady state probability distribution.

The probability distribution $P(x_{1},x_{2},\ldots ,x_{n},t)$ of a dynamical system evolves in time, which is governed by probabilistic diffusion equations. Here $x_{1},x_{2},\ldots ,x_{n}$ represent the concentrations of different molecules in cells. To obtain the probability distribution of the proliferation–quiescence decision system, we follow a self consistent mean field approach [27,31,37,38,39] to split the probability into the products of the individual ones, i.e., $P\left(x_{1},x_{2},\ldots ,x_{n},t\right)∼\prod_{i}^{n}P\left(x_{i},t\right)$ and solve the probability self-consistently.

For a high dimensional system, diffusion equations are hard to be solved directly. Hence, we start from the moment equations. In this work, we assume Gaussian distribution as an approximation, which means we need to calculate two moments, the mean and the variance. When the diffusion coefficient *D* is small, the moment equations can be approximated to [40,41]:

(1) $$\dot{\bar{x}}\left(t\right)=F\left[\bar{x}\left(t\right)\right]$$

(2) $$\dot{\sigma }\left(t\right)=\sigma \left(t\right)A^{T}\left(t\right)+A\left(t\right)\sigma \left(t\right)+2D\left[\bar{x}\left(t\right)\right].$$

Here, x, σ(t), and A(t) are vectors and tensors, and $A^{T}\left(t\right)$ is the transpose of A(t). $\bar{x}\left(t\right)$ represents the mean, σ(t) represents covariance matrix, and the diagonal elements of σ(t) represent the variances of different components. A(t) is the linearization (Jacobian) of the nonlinear system and is evaluated at different stable states (equilibria) as shown in Table S1.

The elements of matrix A are specified as: $A_{\mathrm{ij}}=\frac{\partial F_{i}\left[x\left(t\right)\right]}{\partial x_{j}\left(t\right)}$. Based on these equations, we can solve $\bar{x}\left(t\right)$ and σ(t). Here, we only consider the diagonal elements of σ(t) from the mean field approximation. Therefore, the evolution of probability distribution for each variable can be obtained from the Gaussian approximation:

(3) $$P\left(x,t\right)=\frac{1}{\sqrt{2\pi \sigma (t)}}\exp-\frac{{\left[x-\bar{x}\left(t\right)\right]}^{2}}{2\sigma (t)}$$

Here, $\bar{x}\left(t\right)$ and σ(t) are the solutions of Equations (1) and (2). The probability distribution obtained above corresponds to one steady state or basin of attraction. If the system has multiple steady states, there should be several probability distributions localized at each basin with different variances. Therefore, the total probability is the weighted sum of all these probability distributions. From the mean field approximation, we can extend this formulation to the multidimensional case by assuming that the total probability is the product of each individual probability for each variable. We define $P_{ss}\left(x\right)$ as the total probability distribution in steady states. Based on previous work [27,28,42], one way of calculating the potential landscape is from steady state probability distribution. Therefore, with the total probability at steady state, we can construct the potential landscape by: $U\left(x\right)=-lnP_{ss}\left(x\right)$ [27,42].

### 2.3. Transition Paths

To quantify the transition feasibility between two attractors (cell states), we resort to the transition path theories [32,43,44,45], with which we can calculate the most probable kinetic transition path by minimizing the transition actions from one state to another. A dynamical system in the fluctuating environments can be addressed by:

(4) $$\dot{\bar{x}}\left(t\right)=F\left[\bar{x}\left(t\right)\right]+\zeta .$$

Here, $x=(x_{1}\left(t\right),x_{2}\left(t\right),\ldots ,x_{n}\left(t\right))$ represents the vector of the expression level of proteins or genes. $F[\bar{x}\left(t\right)]$ is the vector for the driving force from the dynamical system. ζ is the Gaussian white noise term. Let *i*, *j* represent the index of different variables (*i*, *j* = 1, 2, 3, 4, …, *n*), where n is the total number of variables in the model; then we have:

(5) $$E[\zeta_{i}]=0,$$

(6) $$E\left[\zeta_{i}\left(t\right)\zeta_{j}\left(t^{\prime }\right)\right]=2D\delta_{ij}\delta \left(t-t^{\prime }\right),$$

where *D* is the constant diffusion coefficient and

(7) $$\left\{\begin{array}{c} \delta_{ij}=1\;for\;i=j,\\ \delta_{ij}=0\;for\;i\neq j. \end{array}\right.$$

In Equation (6), $\delta_{ij}$ means that the correlation of noise for different species (i≠j) is zero, and $\delta (t-t^{\prime })$ is a Dirac delta function, meaning that for one variable, the correlation of noise at different times ($t\neq t^{\prime }$) is zero, i.e., the white noise limit. Following the approaches [32,43,44,45] based on the Wentzell–Freidlin theory [46], the most probable transition path from attractor *i* at time 0 to attractor *j* at time *T*, can be acquired by minimizing the action functional over all possible paths:

(8) $$S_{T}\left[\phi_{ij}\right]=\frac{1}{2}\int_{0}^{T}{\left|\dot{\phi_{ij}}-F\left(\phi_{ij}\right)\right|}^{2}dt;$$

$\phi_{ij}$ is a path from attractor *i* to attractor *j*. The meaning of Equation (8) is that in principle there could be many paths starting from *i* and ending in *j*. However, each path has different actions based on the definition of action in Equation (8). Thus, our purpose is to identify the very path with the minimized action. From the definition of transition action (Equation (8)), it actually minimizes the difference between the velocity along the path ($\dot{\phi_{ij}}$) and the driving force ($F(\phi_{ij})$), integrated with time. That makes sense, because if one always follows the direction of driving force, then the action will be the least. This path is called the minimized action path (MAP). To minimize the transition action, we used the numerical algorithm of the minimized action path from previous work [45].

### 2.4. Processing Single-Cell Data with a K-Means Algorithm

To see how the single-cell data agree with our landscape model, we performed cluster analysis for single-cell data. We obtained the data from Gookin’s work [30]. They found three clusters in the data and classified them as G0, G1, and “cells in other phases” (see Figures 1C and 2 in [30]). We assigned the “cells in other phases” to the S cluster. Then, we used the k-means algorithm (using Matlab) to process the single-cell data and set the number of clusters as three to be consistent with previous experiment in [30]. To further test whether the optimal clustering number of single-cell data is three, we employed the gap statistic method in Matlab’s toolbox [47]. The gap statistic value can be viewed as the difference between the loss of the random sample and the loss of the actual sample. Therefore, higher scores correspond to better clustering. The gap statistic result confirmed that the most appropriate cluster number in the single-cell data is three (Figure S5).

## 3. Results

### 3.1. Landscape and Path for the Proliferation–Quiescence Decision

Based on previous work [12], we constructed a model for the proliferation–quiescence decision, and quantified the corresponding energy landscape [25,27,31,33,48]. For visualization, we picked pRb and p21 as the two coordinates and projected the landscape to a two-dimensional space (Figure 1B). The blue region on the landscape represents lower potential or higher probability while the yellow region represents higher potential or lower probability.

We obtained the three-dimensional (Figure 1B) and two-dimensional (Figure 1C) landscape, respectively. Three attractors emerge on the landscape, which correspond to the G0 state (resting state unable to pass the RP), G1 state (passing the RP, but halted in G1/S checkpoint), and S state (entering the proliferative stage). In this work, we focused on the G0 to G1 and G1 to S transitions and did not consider the transition from S to G2/M phase. The directional arrows connecting each state represent the MAPs among different attractor states. The MAP is the most probable transition path from one attractor to the other. The white arrows represent the transitions from quiescence to proliferation state, and the pink arrows represent the transitions reversing the proliferative process. The landscape with three attractors is supported by three clusters classified as G0, G1, and S in single-cell data [30] by applying the k-means algorithm [49] (Figure 1D). Here, the phospho-Rb axis denotes the phosphorylation level of Rb (Figure 1D), which decreases as pRb increases. To provide additional evidence that there are three clusters in single-cell data, we plotted the pRb vs. DNA and p21 vs. DNA (Figure S4A,B). These results have three clear clusters under the cell cycle decisions. Besides, we employed the gap statistic method to do the analysis [47], which showed that the optimal clustering number for single-cell data is three (Figure S5). On the landscape, the S state is the lowest basin describing the highest occupation probability of cell states (Figure 1B), which happens to match the S cluster with the largest number of cells from single-cell data (Figure 1D). These results indicate that our models agree well with experimental data.

To study cell cycle transitions at the RP and G1/S checkpoints, we monitored the expression profiles of key regulators in temporal order by the MAPs based on the model (Figure 2). In Figure 2, “1” represents the “on state” or activated state, and “0” represents the “off state” or inactivated state. The threshold we assumed here is the average of the highest value and the lowest value during the transition from G0 to S. That is, if the expression level is larger than the threshold, then it is set to be “1.” Otherwise, it is set to be “0.” During the G0->G1 transition, CycE, CycA, and E2F are inactivated, while p53, p21, and Cdh1 are activated (Figure 2A). An obvious change in the G0->G1 transition is that Rb is deactivated, which is consistent with the experimental observation showing notable hyperphosphorylation of Rb in G0->G1 transition [14]. In the G1->G0 transition, a major change is that Rb is accumulated to its threshold (Figure 2B) and causes the G0 quiescence [50], which indicates critical roles of Rb in inducing quiescence.

For the transition from G1 to S, CycE and CycA reach their thresholds after the E2F activation and the p21 degradation (Figure 2C). Rb stays silenced and p53 is turned down in G1->S transition. Emi1 stays at a low level until the late G1 phase, while Cdh1 is highly expressed (Figure 2A,C), which is supported by the experiment [22]. Once the cell cannot maintain its state in S and reverses to the G1 state (S->G1), overexpressed p53 activates p21 and inhibits CycA, CycE, and E2F (Figure 2D). This is consistent with the roles of p53 inducing the G1 cell cycle arrest [17]. Intriguingly, p53 is shut down in G1->G0 transition but kept activated in the S->G1 transition, which suggests that the S->G1 transition might be caused by the cellular DNA damage [13].

We also traced the direct transition path of molecular activation in G0->S (Figure 2E) and S->G0 transition (Figure 2F). In the G0->S transition (Figure 2E), p53 is downregulated first, and followed by the inhibition of Rb, Cdh1, and p21. Then, E2F, CycE and other positive cell cycle regulators are expressed and accumulated. In the S->G0 transition, p53 is upregulated first to stimulate p21 and other negative cell cycle regulators (Figure 2F). Of note, some dual-negative feedback components, such as Rb and E2F, are not synchronized to reach their threshold. The inhibition of CycA and CycE happens before the p21 synthesis, and Emi1 declines quicker than Cdh1 reaching its threshold (Figure 2F). These results suggest that to block proliferation, the activation of P53 and deactivation of CycE and CycA play leading roles. Moreover, the results of kinetic transition paths clearly indicate that Rb controls RP and p21 plays major roles in G1 to S transition (Figure 2A–D).

### 3.2. Rb Controls RP and P21 Governs the G1/S Checkpoint

To further clarify the mechanisms of checkpoints in the cell cycle, we propose that the two saddle points on the landscape characterize the RP and G1/S checkpoints, respectively. Through making perturbations to key regulators, we can discover how different factors influence the landscape (Figure 3A–J and Figures S6–S10). We let the total level of Rb descend gradually by reducing the synthesis rate of Rb (Figure 3A–F). When Rb is at a high level, all cells are trapped in G0 state and cannot initiate proliferation (Figure 3A). As the Rb concentration slightly dilutes, cells gather in the G0 and S states (Figure 3B). A stable G1 state is missing, which suggests that the G1/S checkpoint may lose its efficacy. The downregulation of Rb makes the G1/S checkpoint disappear, indicating their potential to be cancer cells [51,52].

When Rb concentration continues to decrease, the landscape will display a tristable state (Figure 3C,D). The G1 state contains the cells which are blocked on the G1/S checkpoint after going through the RP. The G0 state shrinks with the downregulation of Rb (Figure 3A–D) and disappears after Rb goes below a certain threshold (Figure 3E). The disappearance of G0 state suggests that the function of RP cannot be maintained at low Rb concentration. This provides a quantitative and physical explanation for Rb controlling RP. Finally, as Rb continues to decline, only a stable S basin is left with extremely low concentration of Rb (Figure 3F), signifying that Rb is a negative regulator for G1 to S transition.

We also showed how the landscape changes as the synthesis rate of p21 is perturbed (Figure 3G–J). Highly-activated p21 eliminates the S phase and retains the G0 and G1 phases (bistable state), confirming that p21 controls another checkpoint downstream of the RP (Figure 3G). An appropriate range of p21 synthesis will generate a tristable landscape (Figure 3H). After the level of p21 continues to decrease, the G1 disappears prior to the disappearance of G0 state (Figure 3I). This affirms that p21 is vital to the G1/S checkpoint. Besides, an extremely low level of p21 gets rid of the G0 state, which suggests that p21 might act as a negative regulator and prevent the proliferation in RP (Figure 3J).

The perturbation of E2F has similar effects to the perturbation of Rb (Figure S6A–F), implying their reciprocal roles in the cell cycle [53,54]. A high level of E2F leads to an “S-only” state (Figure S6F), which is consistent with the previous observations in multiple cancer cells [54,55]. The overexpressions of CycA and CycE also accelerate the proliferation (Figures S9F and S10F). However, they might have auxiliary roles in RP compared to their dominant roles in G1/S checkpoint [56], as they are not able to cause a “G0-only” state (Figures S9A and S10A). Low expression level of Emi1 blocks the proliferation (Figure S7A). However, the landscape of Emi1 in high level suggests that the overexpression of Emi1 may cause a mitotic block too (Figure S7F). This phenomenon observed in somatic cells indicates that Emi1 plays more roles than being a positive regulator in early mitosis [22]. A high level of Cdh1 prevents the cell cycle from transiting to the S phase (Figure S6A), which suggests that the cell-cycle commitment might be mediated by the dual-negative feedback switch of Emi1 and Cdh1 [34].

To quantify the transition of the proliferation–quiescence decision, we define the potential barrier (*U*) as the potential difference between the local minimum and the corresponding saddle point [32]. A larger barrier means a more difficult state transition. We also calculated the transition action (*S*) as another measure for the feasibility of each transition [32,57]. A smaller transition action, corresponding to a smaller barrier height, means a easier state transition. The transition action is calculated from the multi-dimensional system directly, and therefore provides a more accurate description than the barrier height.

In Figure 3K–P, we show the transition action changes with the levels of Rb, E2F, and P21. As Rb increases, the transition action of G0->G1 increases and the transition action of G1->G0 decreases (Figure 3K). However, for the G1 to S transition, the transition action does not change significantly as Rb increases (Figure 3L). This is consistent with our previous conclusion that Rb controls the G0->G1 transition (RP). Figure 3M,N shows that E2F has an opposite role for the transition action from G0 to G1, as well as from G1 to S, compared with Rb. This coincides with the notion that E2F and Rb play reciprocal roles, and together control RP [54]. When it comes to the G1/S checkpoint, they both barely influence the S->G1 action (Figure 3L,N). The limited alteration in G1->S compared with the G0->G1 demonstrates that Rb and E2F have more impact on G0->G1 than the G1->S [53]. The quantitative results of Rb and E2F are consistent with previous patterns (Figure 3F and Figure S6F), which support that E2F assists the proliferation, while Rb disturbs it [58].

Differently from E2F and Rb, p21 does not have a notable effect on the transition action (Figure 3O) between G0 and G1. However, the transition action from G1 to S will increase as p21 rises (Figure 3P), indicating that p21 makes it tougher to go through the G1/S checkpoint [50,59]. We noticed that the transition action for S->G1 keeps in a uniform level rather than dial down acutely. This result suggests that the cells in S phase are not sensitive to p21-induced quiescence, which is supported by experimental observations [12,60]. This is also consistent with the transition path results showing that the activation of p21 appears in the later stage of S to G1 transition (Figure 2D). The results of transition actions emphasize that p21 is a major negative regulator for the G1->S transition. Our modeling results based on landscape and paths are supported by in vitro experiments [18,52,61], which gives us inspiration to study more molecular regulatory mechanisms.

### 3.3. Landscape Quantifies the Effects of Growth Factor and DNA Damage

Landscape results illustrate that Rb control the RP and p21 is in charge of the G1/S checkpoint. We further evaluate the roles of growth factor and DNA damage based on landscape topography. To do that, we modeled the depletion of Rb, p21, Cdh1 and compared simulation results with the undisturbed system (Figure 4). Specifically, we set four conditions of each depletion, which correspond to high and low concentration of mitogens, and high and low intensity of DNA damage, individually. We used the growth factor (induced CycD) to reflect the level of the mitogens.

Under the unperturbed condition (Figure 4A–D), the mitogen is necessary for proliferation. Cells without sustaining growth factor signals arrest in G0 state (Figure 4A,C). When the DNA damage is weak, cells are transformed to the S state with sustaining mitogen (Figure 4C,D). However, high intensity of DNA damage will interrupt the proliferation and lead to the G1 arrest (Figure 4B). These results are consistent with previous study showing that a certain threshold of growth factor and the ratio between growth factor and DNA damage control the proliferation [12].

The loss of Rb removes the necessity of the mitogen and invalidates the RP (Figure 4E–H), which is consistent with the landscape results in Figure 3. Cells without abundant growth factor go forward to S state under low intensity of DNA damage (Figure 4C,G), which has been validated experimentally [51]. Despite that fact that RP losses its function, the G1/S checkpoint still responds to the intensity of DNA damage (Figure 4E,F). Regardless of the concentration of the mitogen, the cells will be blocked to the G1 state when suffering from high intensity of DNA damage.

Further, the absence of p21 makes cells less responsive to DNA damage (Figure 4I–L). The concentration of the mitogen affects the proliferation–quiescence decision (Figure 4K,L). We noticed that cells with abundant mitogen proliferate under high intensity of the DNA damage (Figure 4J). This pattern suggests that the G1/S checkpoint is not able to restrain the growth signal when p21 is knocked out, consistently with the landscape results for altering the synthesis rate of p21 (Figure 3G–J). The p21 depletion has minor effects on the G0 basin under a low level of growth factor (Figure 4A,C,I,K) in accordance with the characteristic of p21, which mostly plays roles in G1/S checkpoint (Figure 2 and Figure 5).

Moreover, the removal of another negative cell cycle regulator Cdh1 has no apparent effects on the landscape (Figure 4M–P). The Cdh1-depleted system still responds to DNA damage and the mitogen stimulation. These patterns are similar to the undisturbed system (Figure 4A–D) and consistent with the results of changing synthesis rate (Figure S8F). A previous experiment reported that the depletion of Cdh1 increases the genomic instability and the sensitivity to DNA-damaging agents while not significantly altering the distribution of the various mitotic phases [62]. Landscape results demonstrate that different regulators play diverse functions in the cell cycle. To reveal the subtle mechanisms for proliferation–quiescence decision, we need to evaluate the system in quantitative ways.

### 3.4. The Disparate Roles of Emi1 and Cdh1 in G0 to G1 and G1 to S Transitions

To study the roles of Emi1, Cdh1, and other regulators in the proliferation–quiescence decision, we calculated the transition action as these regulators changed for both the G0 to G1 and G1 to S transitions (Figure 5 and Figures S11–S14). For the G0 to G1 transition, Emi1 increases the action for both the G1->G0 and G0->G1 transitions (Figure 5A). The ratio pattern of Emi1 evinces that the middle level of Emi1 will facilitate the G0 state more than the G1 state (Figure 5B). However, a certain degree of Cdh1 reduces the action for both the G0->G1 and G1->G0 transitions when it comes to the RP (Figure 5E). The ratio pattern also suggests that Cdh1 might play positive role in the RP (Figure 5F), which reminds us that Cdh1 is highly expressed while Emi1 is inhibited until the late G1 phase after the mitosis [22] (Figure 2). These results suggest that Emi1—the early mitotic inhibitor—might have the negative effect on the RP. One possible regulatory mechanism of Emi1 and Cdh1 could be proposed based on the network topology (Figure 1A). A high level of Cdh1 inhibits CycA and thus relieves CycE. Therefore Cdh1 promotes the E2F-dependent upregulation of CycE, which is supported by previous experiments showing that activated Cdh1 does not significantly affect the stability of E2F proteins but rather promotes the E2F transcription [63]. Another possible mechanism is that induction of Cdh1 leads to the decline of p27 (CKI) and increases the Cdk2 activity by accumulation of Skp2 [63]. In Emi1-depleted cells, the re-replication will not be suppressed until Cdh1 is depleted [64]. As the inhibitor of Cdh1, Emi1 blocks re-replication to preserve the genome integrity, which suggests that Emi1 might be a latent depressor for protecting the genome stability and maintaining the functional balance of cells after mitosis.

The G1->S transition action (red line) is higher than the S->G1 action (blue line) when Emi1 is at a low expression level (Figure 5C), implying that the G1->S transition is more difficult than the S->G1 transition with a low concentration of Emi1. When the expression level of Emi1 is high, the G1->S action (red line) is lower than the S->G1 action (blue line), meaning that the G1->S transition is easier than the S->G1 transition with a high concentration of Emi1 (Figure 5C). The ratio of the G1->S action to the S->G1 action declines sharply (Figure 5D). These results reflect that a high level of Emi1 aids the G1->S more than the S->G1 transition, which is consistent with previous findings [34,50]. On the other hand, the elevation of Emi1 makes both G1->S action and S->G1 action larger, and the S->G1 action climbs faster than the G1->S action. Even though the action ratio between G1->S and the S->G1 declines with the Emi1 increment, the absolute value of the action still increases. This trend suggests that overexpressed Emi1 might balk the proliferation (Figure 5C), which is consistent with previous patterns that S state disappears with extremely high concentration of Emi1 (Figure S7). This phenomenon was reported in somatic cells [22], and it was found that the overexpression of Emi1 in vivo increased the chromosome instability [65].

In biological systems, Emi1 stays at an extremely low level during the G1 phase, while Cdh1 expresses at a high level [66]. Acute accumulation of Emi1 at the end of G1 phase inactivates Cdh1 and promotes the S phase (Figure 2C), which is considered a symbol of the G1/S checkpoint [34]. These observations for Emi1 in the organism support our quantitative analysis of landscape models.

The inflation of Cdh1 makes the G1->S action raised, but its impact on S->G1 action is marginal (Figure 5G). The ratio of the G1->S action to the S->G1 action increases with Cdh1 (Figure 5H). These results indicate that Cdh1 prevents the G1->S transition and hardly affects the S->G1 transition. Cdh1 is characterized as an inhibitor at the G1/S checkpoint; Emi1 is an activator.

### 3.5. Global Sensitivity Analysis on Key Regulations and Network Structure

To investigate different regulators on the dynamics of the proliferation–quiescence decision, we performed a global sensitivity analysis on model parameters. The concept of “global stability” is opposed to “local stability”, which corresponds to the local sensitivity analysis. The aim of local sensitivity analysis is to search the local stability condition under the perturbations. That is, the sensitivity analysis based on local stability cannot provide the information on the relative stability for different stable states (basins).

However, the local stability analysis cannot provide information on global stability. The global stability is important for multistable cell fate decision systems, as it can quantify the relative stability for different stable cell states. Here, we use the global stability analysis to study how the relative stable states of a tristable system and transitions among them are affected by perturbations of genes or gene regulation.

We changed each activated or inhibitory constant respectively (characterizing the intensities of individual biochemical reactions) to see how the transition actions between G0 and G1 (Figure 6A,B) and between G1 and S (Figure 6C,D) are influenced. We increased (Figure 6A,C) and decreased (Figure 6B,D) each of the 23 parameters by 0.5% or 5%, individually. Of note, the global sensitivity analysis we performed was based on the transition action between attractors, which were directly calculated from the multi-dimensional system. The complete results for sensitivity analysis are shown in the Supporting Information (Figures S15–S19).

The upregulation of the synthesis rate of E2F and cyclins has a significant impact on the transition between G0 and G1 (Figure 6A,B). However, multiple E3 ligases mediate the degradation of E2F and Cyclins, including the $CRL4^{Cdt2}$ (Cdt2) and the $SCF^{skp2}$ (skp2) [61,67]. However, someone reported that skp2 and Cdt2 also degrade the CKIs, such as p21 and p27 [68,69]. Therefore, the effects of skp2 and Cdt2 on early mitosis remain unclear. Our landscape results help to reconcile their roles on proliferation–quiescence decisions (Figure 6). In the RP skp2 facilitates the G0->G1 transition and inhibits the G1->G0 transition (Figure 6A,B), which is attributed to the skp2-mediated Rb degradation that is promoted by E2F [70]. In the G1/S checkpoint, skp2 facilitates the G1->S transition and inhibits the S->G1 transition (Figure 6C), which is supported by the skp2-depletion experiment [12]. On the other hand, Cdt2 shows the opposite characteristics to skp2 for the cell state transition, even though they are both E3 ligases. Cdt2 adds up to the G0->G1 action and reduces the G1->G0 action, signifying that Cdt2 prevents the G0->G1 transition (Figure 6A,B). Cdt2 lifts up the G1->S action and cuts down the S->G1 action, reflecting Cdt2 prevents the G1->S transition and facilitates the S->G1 transition (Figure 6C). The Cdt2-mediated E2F degradation during S phase has been observed in *Drosophila* cells [71]. Some studies reported that Cdt2 promotes the degradation of skp2, which is consistent with their opposite roles in proliferation–quiescence decisions [56].

The p53-dependent p21 synthesis has a negative impact on G0->G1 and G1->S transitions (Figures S17 and S18). By inhibiting Cdks, p21 mediates the cell cycle arrest in response to DNA damage [59]. The action patterns of CycA and CycE demonstrate that they are not able to maintain pRb hyperphoshporylation in the RP (Figures S11B and S13B), which is different from the formal hypothesis [53,54] and supported by a recent work [72]. They reported that CycE and CycA can sustain Rb hyperphosphorylation only from the start of the S phase while CycD performs this job in the RP. CycD increases the G1->G0 action and reduces the G0->G1 action (Figure 6A,B) by inactivating pRb, thereby promoting the G0->G1 transition (Figure 6A,B). CycD reduces both G1->S action and S->G1 action (Figure 6C), making the migration between G1 and S states more convenient. The overexpression of CycD has been observed in multiple forms of cancer [6]. The CycE-mediated Rb phosphorylation enhances the G1->S transition and inhibits S->G1 transition respectively (Figure 6A,B). Our results suggested that the simultaneous overexpression of CycD and CycE might have a drastic impact on the G1->S transition. A previous study recorded that either overexpressed CycD or overexpressed CycE only moderately accelerates the G1->S transition, while the overexpression of them together brings about a remarkable acceleration for the G1->S transition [13].

To study the influence of the network structure changes on the global stability of the system, we randomly removed and reassigned 20 edges, individually (Tables S2 and S3). Here, we focus on the change of the number of stable states and the energy landscape shape. We show the change of stable states for randomly deleting 20 edges (Table S2) and for randomly reassigning 20 edges (Table S3), individually. To visualize the effect of network structure perturbation on the system stability, we show some examples of landscape change for link deletion and link reassignment perturbations, individually (Figures S20 and S21). In Figure S20, random edge deletions cause landscape alteration. Deleting the inhibitory effect of E2F on Rb leads to a “G0-only” state (Table S2 and Figure S20A). Deleting the inhibitory effect of p53 on DNA damage leads to a “G0” and “G1” bistable state (Table S2 and Figure S20B). Deleting the inhibitory effect of Cdh1 on CycA leads to three stable states (Table S2 and Figure S20C). In Figure S21, random edge reassignments also cause landscape alteration. Changing the inhibitory regulation of Rb on E2F to the inhibitory regulation of p21 on E2F leads to a “G0-only” state (Table S3 and Figure S21A). Changing the inhibitory regulation of CycE on p21 to the inhibitory regulation of CycE on Emi1 leads to “G0” and “G1” states (Table S3 and Figure S21B). Changing the inhibitory regulation of CycA on CycE to the inhibitory regulation of CycA on E2F leads to three stable states (Table S3 and Figure S21C).

Our results show that the perturbations on the network structure will affect the number of stable states (Tables S2 and S3) and the landscape shape (Figures S20 and S21). That is, the phase change can happen with structure perturbations, e.g., from tristable to bistable or monostable state. In the meantime, we also found that the three stable states for G0, S, and G1 cell states still exist with the structure perturbations, despite the phase transition (Tables S2 and S3). This indicates that the tristable landscape results from our model are robust to structure perturbations to a certain degree. This also provides a potential way to identify key structural elements critical for the function of the cell cycle.

## 4. Discussion

The cell cycle has been described as a series of deterministic events with deterministic functions [12], but the global stability and effects of the stochasticity on this process, e.g., proliferation–quiescence decision process, are fully understood [50]. In this study, we focused on the transition process in the cell cycle to reveal the underlying stochastic dynamical mechanism of the proliferation–quiescence decision using a landscape and path approach. Three attractors emerge on the landscape, which correspond to G0, G1, and S phases in the cell cycle. We propose that the proliferation–quiescence decision in early mitosis can be understood as a tristable switch, which agrees well with single-cell data [30]. In the landscape view, the two saddle points on the landscape correspond to the RP and G1/S checkpoints, respectively, which provides new explanations for the mechanism of checkpoints of cell cycle.

The transition from quiescence to proliferation state takes two steps consisting of the transition from G0 to G1 and the transition from G1 to S. To enter the proliferation stage, a cell in G0 quiescence state first turns off Rb and accumulates E2F to cross the RP (the saddle point between G0 and G1), and then turns off p21 to cross the G1/S checkpoint (the saddle point between G1 and S). We inferred the activation order of key regulators involved in the G0 to G1 and the G1 to S transition by calculating the transition path. The most important event in the transition between G0 and G1 is Rb switching on/off. In the G1->S transition, CycE and CycA meet their thresholds after E2F activation and p21 degradation. Emi1 reaches its threshold during the late G1 phase [34]. In the S->G1 transition, p53 activates p21 and inhibits CycA, CycE, and E2F, which suggests that the S->G1 transition might be caused by the cellular DNA damaged [69].

By calculating the transition actions and the potential barriers based on landscape topography, we can quantitatively measure the feasibility of transitions among G0, G1, and S phase. These quantitative results confirm that Rb controls the RP and p21 controls the G1/S checkpoint. We further verified these conclusions by depleting Rb, p21, and Cdh1. The cells without sustaining growth factor arrest in G0 when the DNA damage is weak. The Rb depletion removes the necessity of the mitogen and invalidates the RP. The absence of p21 makes cells less responsive to DNA damage, which means that the G1/S checkpoint is not able to restrain the growth signal.

The transition action patterns of Emi1 and Cdh1 reveal their unique roles on the proliferation–quiescence decision. Emi1 might have negative effects, and Cdh1 might play positive roles on the RP. Two mechanisms may be proposed and supported by experiments [63]. One is that a high level of Cdh1 relieves CycE by repressing CycA, and thus promotes the E2F transcription. Another possible mechanism is that the induction of Cdh1 leads to the decline of p27 (CKI) and increases the Cdk2 activity through Skp2. As the inhibitor of Cdh1, Emi1 might be a latent depressor to protect the genome stability. A high level of Emi1 aids the G1->S more than the S->G1 transition. Even though the action ratio between G1->S and the S->G1 decreases with the Emi1 increment, the absolute value of the action still increases, which suggests that the overexpression of Emi1 might block the proliferation. This phenomenon was reported in somatic cells [22] and supported by recent experiments [73].

To investigate how the cell cycle dynamics are influenced by different regulators, we performed a global sensitivity analysis on model parameters. Our global sensitivity analysis reveals that the synthesis rate of Rb, the synthesis rate of E2F, and the self-activation rate of E2F have strong impacts on the G0->G1 transition. We found that skp2 facilitates the G0->G1 transition and inhibits the G1->G0 transition. While in the G1/S checkpoint, skp2 promotes the G1->S transition and inhibits the S->G1 transition. Cdt2 shows an opposite characteristics on the cell state transition compared with skp2 [56]. Cdt2 prevents the G0->G1 transition and G1->S transition while facilitating the S->G1 transition. The p53-dependent p21 synthesis has a negative impact on G0->G1 and G1->S transition. The action patterns of the CycA and CycE demonstrate that they cannot sustain pRb hyperphoshporylation on the RP [72]; CycD performs that job. CycD inactivates pRb significantly and promotes the G0->G1 transition. However, the CycD-mediated pRb phosphorylation reduces both G1->S action and S->G1 action, which makes the migration between G1 and S states more convenient. The CycE-mediated Rb phosphorylation enhances the G1->S transition and inhibits S->G1 transition specifically. Our results suggested that the overexpressing CycD and CycE simultaneously might have a drastic impact on the G1->S transition [13].

We need to stress that our conclusions are not only based on two-dimensional results, although we used pRb and p21 as coordinates here for the landscape. In fact, our results for transition paths between attractors were calculated by minimizing the transition actions based on the complete 20-dimensional model (Figure 3K–P, Figure 5 and Figure 6). The landscape for pRb and p21 is a two-dimensional projection for the complete 20-dimensioanl landscape for visualization. We picked pRb and p21 as coordinates as they are important in cell fate decision since they manage the functions of different checkpoints. We also showed the landscape using other pairs of genes as coordinates (see Figures S2 and S3).

Our results are novel in the following three aspects. First, previous cell cycle models mostly focused on local stability by deterministic ODEs. However, the global stability is hard to be addressed only from deterministic models. Here, we quantified the stochastic transitional dynamics of the proliferation–quiescence decision by quantifying the landscape and calculating the kinetic transition path. Through landscape models, we identified three attractors on landscape corresponding to the G0, G1, and S phases and two saddle points corresponding to restriction point (RP) and G1/S checkpoint. This provides a global view for the stochasticity and dynamical transitions of proliferation–quiescence decision, and a quantitative explanation for RP and G1/S checkpoints. Second, previous work used bistable model to describe these three states [12]. There is evidence showing that G1 and S are not one single state [50]. Additionally, single-cell data suggest the possible tristability [30]. Therefore, we believe that a model with three stable cell states can better fit qualitative and quantitative data [12,30]. Third, we provide some biological predictions, which can be tested in future experiments. For example, our models predict that a medium level of Emi1 promotes G1 to S transition while Cdh1 inhibits G1 to S transition. Interestingly, both high and low levels of Emi1 prevent the G1 to S transition. Emi1 has a negative effect on RP and facilitates G0 quiescence, whereas Cdh1 play positive roles in RP. As we know, the roles of Emil1 and Cdh1 in the cell cycle remain challenging to quantify. Our predictions based on the modeling provide new clues for clarifying the critical roles of these two factors in the cell cycle.

The landscape view provides new explanations for the mechanisms of checkpoints. Specifically, the two saddle points on the landscape can correspond to the RP and G1/S checkpoints, respectively. We found that a high level of Rb annihilates both G1 and S states, whereas a high level of p21 exterminates S state and preserves G0 and G1 states. These results indicate that Rb controls the transition of RP and that p21 is responsible for the G1/S checkpoint [35]. An intriguing result is that both high and low levels of Emi1 eliminate the S state, demonstrating that a suitable range of Emi1 might be necessary for proliferation. This coincides with recent experiments showing that the overexpression of Emi1 in mesenchymal stem cells promotes migration and differentiation rather than proliferation [73]. We found that the transition action of the G0->G1 transition increases as E2F decreases or as Rb increases, suggesting the reciprocal roles of Rb and E2F on RP. The transition action of the G1->S transition increases as CycE and CycA decrease or as p21 increases, suggesting the critical roles of P21 in G1/S checkpoints. Surprisingly, the transition action of the G0->G1 transition slightly increases as CycA and CycE increase, implying that they are not sustained to hyperphosphorylate pRb on the RP [72]. We found that a moderate degree of Emi1 facilitates the G1->S transition, whereas Cdh1 prevents it. However, Emi1 inhibits the G0 to G1 transition while Cdh1 promotes it. Finally, to explore how the other factors influence cell cycle, we performed a global sensitivity analysis on the landscape for different regulators.

The transition action and barrier height based on landscape topography provide quantitative measures for the global stability and transition dynamics of gene network system. In the landscape view, cells need two steps to enter proliferation stage; i.e., first make a transition from G0 to G1 and then make a transition from G1 to S. In this progression process, two checkpoints play critical roles, including RP governed by Rb, and G1/S checkpoint controlled by p21. In this picture, the RP can be understood as the saddle point between G0 and G1 basin, and the G1/S checkpoint can be understood as the saddle point between G1 and S basin. To enter the proliferation stage, a cell in G0 resting state first turns off Rb (turn on E2F) to cross the first barrier (RP), finishing the G0 to G1 transition, and then turns off p21 to cross the second barrier (G1/S checkpoint), finishing the G1 to S transition. Therefore, the landscape results provide a global and new picture for proliferation–quiescence decisions, and an explanation for the mechanism of checkpoints from the physical perspective.

We need to stress that current results provide one possible scenario in the huge parameter space of the cell cycle network. It remains challenging to perform an exhaustive parameter search in the whole state space and an explicit network structure analysis [74,75]. It will be also of great interest to develop more inclusive models by incorporating G2 and M phases, and study the dynamical mechanism of fate transitions in the cell cycle. For example, in previous work, Gérard et al. developed a mammalian cell cycle model which completely describes the cell division cycle, including the restriction point and the G1/S transition, with the influences of growth factors [10]. Based on this model, we have uncovered the potential and flux landscape, and proposed a physical explanation for cell cycle checkpoints [27]. Our work provides a general landscape view of the stochasticity and dynamics in cell state transition and facilitates our mechanistic understanding of proliferation–quiescence decisions in early mitosis.

## Supplementary Materials

The following are available online at https://www.mdpi.com/2077-0383/9/8/2582/s1. Table S1. Stable steady states for tristable system. Table S2. Random edge deletion. Table S3. Random edge reassignment. Figure S1. A complete influence diagram of the the early cell-cycle model. Figure S2. The landscape is displayed using different pairs of components. Figure S3. Cell quiescence-proliferation fate decision landscape. Figure S4. Single cell data supporting three stable cell states. Figure S5. Gap statistic results for the single cell data. Figure S6. Landscape changes as the synthesis rate of E2F changes. Figure S7. Landscape changes as the synthesis rate of Emi1 changes. kSyE1 is the synthesis rate of Emi1. Figure S8. Landscape changes as the concentration of Cdh1 changes. C1t is the total concentration of Cdh1. Figure S9. Landscape changes as the synthesis rate of CycA changes. kSyCa is the synthesis rate of CycA. Figure S10. Landscape changes as the synthesis rate of CycE changes. Figure S11. Effect of CycA (A–D) on the transition action (S) and barrier (U) between G0 and G1. Figure S12. Effect of CycA (A–D) on the transition action (S) and barrier (U) between G1 and S. Figure S13. Effect of CycE (A–D) on the transition action (S) and barrier (U) between G0 and G1. Figure S14. Effect of CycE (A–D) on the transition action (S) and barrier (U) between G1 and S. Figure S15. Sensitivity analysis for the 11 parameters, which control the synthesis of regulators, on the transition action of G0->S (A), S->G0 (B). Figure S16. Sensitivity analysis for the 12 parameters, which control the degradation of regulators, on the transition action of G0->S (A), S->G0 (B). Figure S17. Sensitivity analysis for the 22 parameters, which control the interaction between regulators, on the transition action of G0->G1 (A), G1->G0 (B). Figure S18. Sensitivity analysis for the 22 parameters, which control the interaction between regulators, on the transition action of G1->S (A), S->G1 (B). Figure S19. Sensitivity analysis for the 22 parameters, which control the interaction between regulators, on the transition action of G0->S (A), S->G0 (B). Figure S20. Random edge deletion causes landscape alteration. Figure S21. Random edge reassignment causes landscape alteration. (A) Change the inhibitory effect of Rb on E2F to the inhibitory effect of p21 on E2F.

## Appendix A. Mathematical Modeling

### Appendix A.1. Restriction Point and G1/S Checkpoint

$\frac{d(Rb)}{dt}=Rb_{t}\cdot k_{Rb,Cd}^{Ph}-Rb\cdot \left(k_{Rb}^{Dp}+k_{Rb,Cd}^{Ph}\cdot Cd+k_{Rb,Ce}^{Ph}\cdot Ce+k_{Rb,Ca}^{Ph}\cdot Ca\right)+(k_{RbE2F}^{Ds}+$

$k_{E2F}^{De}-k_{Rb}^{Dp})\cdot RbE2F-k_{RbE2F}^{As}\cdot Rb\cdot E2F$
$r_{Rb}^{Ph}=k_{Rb,Cd}^{Ph}\cdot Cd+k_{Rb,Ce}^{Ph}\cdot Ce+k_{Rb,Ca}^{Ph}\cdot Ca$
$Rb_{t}=Rb_{u}+Rb_{p}=Rb+RbE2F+Rb_{p}$
$\frac{d(E2F_{t})}{dt}=r_{E2F}^{Sy}-k_{E2F}^{De}\cdot E2F_{t}$
$E2F_{t}=E2F+RBE2F$
$\frac{d(E2F)}{dt}=r_{E2F}^{Sy}-k_{E2F}^{De}\cdot E2F-k_{RbE2F}^{As}\cdot Rb\cdot E2F+\left(k_{RbE2F}^{Ds}+r_{Rb}^{Ph}\right)\cdot RbE2F$
$r_{E2F}^{Sy}=k_{E2F}^{Sy}+k_{E2F,E2F}^{Sy}\frac{E2F}{j_{E2F}^{Sy}+E2F}$

### Appendix A.2. Synthesis and Degradation of Cyclin and the Inhibition of Emi1 to APC/C Cdh1

$E1_{t}=E1+E1C1$

$\frac{d(E1)}{dt}=k_{E1}^{Sy}\cdot E2F-k_{E1}^{De}\cdot E1+\left(k_{C1}^{Ph}+k_{C1,Ce}^{Ph}\cdot Ce+k_{C1,Ca}^{Ph}\cdot Ca\right)\cdot E1C1-k_{E1C1}^{As}\cdot$
$E1\cdot C1+k_{E1C1}^{Ds}\cdot E1C1$
$r_{Ce}^{De}=k_{Ce}^{De}+k_{Ce,Ca}^{De}\cdot Ca$
$r_{Ca}^{De}=k_{Ca}^{De}+k_{Ca,C1}^{De}\cdot C1$
$r_{C1}^{Ph}=k_{C1}^{Ph}+k_{C1,Ce}^{Ph}\cdot Ce+k_{C1,Ca}^{Ph}\cdot Ca$
$\frac{d(E1C1)}{dt}=-r_{C1}^{Ph}\cdot E1C1+k_{E1,C1}^{As}\cdot E1\cdot C1-\left(k_{E1,C1}^{Ds}+k_{E1,C1}^{De}\right)\cdot E1C1$
$C1_{t}=C1+C1_{p}+E1C1$

### Appendix A.3. Activity of P21 and the Inhibition on Cdk2

$\frac{d(Ce)}{dt}=k_{Ce}^{Sy}\cdot E2F-\left(k_{Ce}^{De}+k_{Ce,Ca}^{De}\cdot Ca\right)\cdot Ce-k_{CyP21_{t}}^{As}\cdot \left(P21_{t}-CeP21-CaP21-Pcna_{i}-Rc_{i}\right)\cdot$

$Ce+(k_{CyP21_{t}}^{Ds}+k_{P21_{t}}^{De}+k_{P21_{t}Cy}^{De}\cdot skp2\cdot \left(Ce+Ca\right)+k_{P21_{t}RC_{a}}^{De}\cdot Cdt2\cdot Rc_{a})\cdot CeP21$
$\frac{d(Ca)}{dt}=k_{Ca}^{Sy}\cdot E2F-\left(k_{Ca}^{De}+k_{Ca,C1}^{De}\cdot C1\right)\cdot Ca-k_{CyP21_{t}}^{As}\cdot \left(P21_{t}-CeP21-CaP21-Pcna_{i}-Rc_{i}\right)\cdot$
$Ca+(k_{CyP21_{t}}^{Ds}+k_{P21_{t}}^{De}+k_{P21_{t}Cy}^{De}\cdot skp2\cdot \left(Ce+Ca\right)+k_{P21_{t}RC_{a}}^{De}\cdot Cdt2\cdot Rc_{a})\cdot CaP21$
$\frac{d(P21_{t})}{dt}=k_{P21}^{Sy}+k_{P21,P53}^{Sy}\cdot P53-r_{P21}^{De}\cdot P21_{t}$
$\frac{d(CeP21)}{dt}=k_{CyP21}^{As}\cdot P21\cdot Ce-\left(k_{CyP21}^{Ds}+r_{Ce}^{De}+r_{P21}^{De}\right)\cdot CeP21$
$\frac{d(CaP21)}{dt}=k_{CyP21}^{As}\cdot P21\cdot Ca-\left(k_{CyP21}^{Ds}+r_{Ca}^{De}+r_{P21}^{De}\right)\cdot CaP21$
$r_{P21}^{De}=k_{P21}^{De}+k_{P21,Cy}^{De}\cdot Skp2\cdot \left(Ce+Ca\right)+k_{P21,Rc_{a}}^{De}\cdot Cdt2\cdot Rc_{a}$
$P21_{t}=P21+CeP21+CaP21+Pcna_{i}+Rc_{i}$
$Ce_{t}=Ce+CeP21$
$Ca_{t}=Ca+CaP21$

### Appendix A.4. Activation of DNA Replication, DNA Damage and Repair

$\frac{d(Rc)}{dt}={-}_{Rc}^{Ph}\cdot Rc+k_{Rc}^{Dp}\cdot Rc_{p}-r_{Rc}^{Ds}\cdot Rc$

$\frac{d(C1)}{dt}=-r_{C1}^{Ph}\cdot C1+k_{C1}^{DP}\cdot C1_{p}-k_{E1C1}^{As}\cdot E1\cdot C1+\left(k_{E1C1}^{Ds}+k_{E1,C1}^{De}\right)\cdot E1C1$
$r_{Rc}^{Ph}=k_{Rc}^{Ph}\frac{{\left(Ce+Ca\right)}^{n}}{{\left(j_{cy}\right)}^{n}+{\left(Ce+Ca\right)}^{n}}$
$r_{Rc}^{Ds}=H\left(DNA-1\right)$
$\frac{d(Rc_{p})}{dt}=r_{Rc}^{Ph}\cdot Rc-k_{Rc}^{Dp}\cdot Rc_{p}-k_{RcPc}^{As}\cdot \left(Pcna_{a}+Pcna_{i}\right)\cdot Rc_{p}+k_{RcPc}^{Ds}\cdot \left(Rc_{a}+Rc_{i}\right)-r_{Rc}^{Ds}\cdot Rc_{p}$
$\frac{d(Rc_{a})}{dt}=-k_{PcP21}^{As}\cdot P21\cdot Rc_{a}+\left(k_{PcP21}^{Ds}+r_{P21}^{De}\right)\cdot Rc_{i}+k_{RcPc}^{As}\cdot Pcna_{a}\cdot Rc_{p}-k_{RcPc}^{Ds}\cdot Rc_{a}-r_{Rc}^{Ds}\cdot Rc_{a}$
$\frac{d(Rc_{i})}{dt}=k_{PcP21}^{As}\cdot P21\cdot Rc_{a}-\left(k_{PcP21}^{Ds}+r_{P21}^{De}\right)\cdot Rc_{i}+k_{RcPc}^{As}\cdot Pcna_{i}\cdot Rc_{p}-k_{RcPc}^{Ds}\cdot Rc_{i}-r_{Rc}^{Ds}\cdot Rc_{i}$
$\frac{d(Pcna_{a})}{dt}=k_{Pc}^{Im}-k_{PcP21}^{As}\cdot P21\cdot Pcna_{a}+\left(k_{PcP21}^{Ds}+r_{P21}^{De}\right)\cdot pcna_{i}-\left(k_{Pc}^{Ex}+k_{RcPc}^{As}\cdot Rc_{p}\right)\cdot Pcna_{a}+$
$\left(k_{RcPc}^{Ds}+r_{Rc}^{Ds}\right)\cdot Rc_{a}$
$\frac{d(Pcna_{i})}{dt}=k_{PcP21}^{As}\cdot P21\cdot Pcna_{a}-\left(k_{PcP21}^{Ds}+r_{P21}^{De}\right)\cdot pcna_{i}-\left(k_{Pc}^{Ex}+k_{RcPc}^{As}\cdot Rc_{p}\right)\cdot Pcna_{i}+$
$\left(k_{RcPc}^{Ds}+r_{Rc}^{Ds}\right)\cdot Rc_{i}$
$\frac{d(DNA)}{dt}=k_{DNA}^{Sy}\cdot Rc_{a}$
$\frac{d(Dam)}{dt}=k_{Dam}^{Ge}+k_{Dam,Rca}^{Ge}\cdot Rc_{a}-r_{Dam}^{Re}\cdot Dam$
$\frac{d(P53)}{dt}=k_{P53}^{Sy}-r_{P53}^{De}\cdot P53$
$r_{Dam}^{Re}=k_{Dam}^{Re}+k_{Dam,P53}^{Re}\frac{P53}{j_{Dam}+Dam}$
$r_{P53}^{De}=\frac{k_{P53}^{De}}{j_{P53}+Dam}$

## Appendix B. Model Parameters

**Table A1:** Parameters for tristable system.

		Value	Value		
Parameter	Description	Used in	from	Unit	Reference
		This Work	Ref. [12]		
kSyCa	constitutive CycA synthesis	0.008	0.02	1/min	estimated
kSyCe	constitutive CycE synthesis	0.004	0.01	1/min	estimated
kSyE2F	constitutive E2F synthesis	0.0642	0.03	AU/min	estimated
kSyE1	constitutive Emi1 synthesis	0.005	0.005	1/min	[12]
kSyP21	constitutive p21 synthesis	0.002	0.002	AU/min	[12]
kSyP53	constitutive p53 synthesis	0.05	0.05	AU/min	[12]
C1t	total $APC/C^{Cdh1}$ level	1	1	AU	[12]
Rbt	total Rb level	5.58	5	AU	estimated
Cd	relative CycD:Cdk4/6 level	0.65	0.65	AU	[12]
Cdt2	relative $CRL4^{Cdt2}$ level	1	1	-/min	[12]
skp2	relative $SCF^{skp2}$ level	1	1	-/min	[12]
kDeCa	constitutive CycA degradation	0.015	0.01	1/min	estimated
kDeCe	constitutive CycE degradation	0.006	0.004	1/min	estimated
kDeE2F	constitutive E2F degradation	0.05	0.05	1/min	[12]
kDeE1	constitutive Emi1 degradation	0.0025	0.0005	1/min	estimated
kDeP21	constitutive p21 degradation	0.0025	0.0025	1/min	[12]
kDeP53	constitutive p53 degradation	0.05	0.05	AU/min	[12]
kDeCaC1	$APC/C^{Cdh1}$-mediated CycA degradation	3	2	1/(AU·min)	estimated
kDeCeCa	CycA:Cdk2-mediated CycE degradation	0.03	0.015	1/(AU·min)	estimated
kDeE1C1	$APC/C^{Cdh1}$-mediated Emi1 degradation	0.005	0.005	1/min	[12]
kDsCyP21	dissociation of cyclin:Cdk2:p21 complexes	0.05	0.05	1/min	[12]
kDsE1C1	dissociation of Emi1:$APC/C^{Cdh1}$ complexes	0.01	0.01	1/min	[12]
kDsRbE2F	dissociation of Rb:E2F complexes	0.005	0.005	1/min	[12]
kPhRbCa	CycA:Cdk2-mediated Rb phosphorylation	0.18	0.3	1/(AU·min)	estimated
kPhRbCd	CycD:Cdk4/6-mediated Rb phosphorylation	0.2	0.2	1/(AU·min)	[12]
kPhRbCe	CycE:Cdk2-mediated Rb phosphorylation	0.18	0.3	1/(AU·min)	estimated
kAsRbE2F	association of Rb and E2F	18	5	1/(AU·min)	estimated
kAsE1C1	association of Emi1 and $APC/C^{Cdh1}$	10	10	1/(AU·min)	[12]
kPhC1	Constitutive $APC/C^{Cdh1}$ phosphorylation	0.01	0	1/min	estimated
kPhC1Ca	CycA:Cdk2-mediated $APC/C^{Cdh1}$ phosphorylation	1	1	1/(AU·min)	[12]
kPhC1Ce	CycE:Cdk2-mediated $APC/C^{Cdh1}$ phosphorylation	0.01	0.01	1/(AU·min)	[12]
kAsCyP21	association of p21 and cyclin:Cdk2	10	10	1/(AU·min)	[12]
kSyE2FE2F	E2F-dependent E2F synthesis	0.06	0.04	AU/min	estimated
kDeP21Cy	cyclin:Cdk2-mediated p21 degradation	0.01	0.007	1/(AU·min)	estimated
kDeP21RCa	$RC_{a}$-mediated p21 degradation	1	0.01	1/(AU·min)	[12]
kDpC1	dephosphorylation of $APC/C^{Cdh1}$	0.05	0.05	1/min	[12]
kDpRb	dephosphorylation of Rb	0.06	0.05	1/min	estimated
kDpRc	dephosphorylation of primed RCs	0.05	0.05	1/min	[12]
kPhRc	cyclin:Cdk2-mediated priming of RCs	0.1	0.1	1/min	[12]
kSyP21P53	p53-dependent p21 synthesis	0.008	0.008	1/min	[12]
kSyDNA	DNA synthesis by active RCs	0.0093	0.0093	1/min	[12]
kGeDam	replication-independent DNA damage	0.001	0.001	AU/min	[12]
kGeDamRCa	replication-dependent DNA damage	0.012	0.012	1/min	[12]
kReDam	P53-independent DNA damage repair	0.001	0.001	1/min	[12]
kReDamP53	P53-dependent DNA damage repair	0.005	0.005	1/min	[12]
jCy	Cdk2 threshold for RC priming	1.8	1.8	AU	[12]
jDam	DNA damage threshold for repair	0.5	0.5	AU	[12]
jP53	inhibition constant of p53 degradation	0.01	0.01	AU	[12]
jSyE2F	Michealis-Menten constant for E2F synthesis	0.2	0.2	AU	[12]
kAsPcP21	association of PCNA and p21	100	100	1/(AU·min)	[12]
kAsRcPc	association of primed RCs and PCNA	0.01	0.01	1/(AU·min)	[12]
kDsPcP21	dissociation of PCNA:p21 complexes	0.01	0.01	1/min	[12]
kDsRcPc	dissociation of $RC_{p}$:PCNA complexes	0.001	0.001	1/min	[12]
kExPc	PCNA export from the nucleus	0.006	0.006	1/min	[12]
kImPc	PCNA import into the nucleus	0.003	0.003	AU/min	[12]
n	hill coefficient for priming of RCs	6	6	0.29	[12]

RC pre-replication complexes; $RC_{a}$ active form of replication complexes. $RC_{I}$ inactive form of replication complexes; $RC_{p}$ primed replication complexes.
