# Supplementary material for: Quantifying the Landscape and Transition Paths for Proliferation–Quiescence Fate Decisions
Source: J Clin Med. 2020 Aug 10;9(8):2582. doi: 10.3390/jcm9082582 (PMC7466041; doi:10.3390/jcm9082582)
Supplement: Supplementary file 1 [file jcm-09-02582-s001.pdf]

# Quantifying the Landscape and Transition Paths for Proliferation-Quiescence Fate Decision

## Supplementary Results

### Descriptions of model parameters

Fig. S1 gives the complete diagram for the whole model. We set the model parameters according to the following criteria:

1. We set most of parameter values of the model by following previous work [1].

2. Previous experimental work suggests that the model with three attractors fits the single-cell data better than the model with two attractors (Fig. S3 and S4) [2]. So, one criteria here is to search the appropriate parameters that can generate three attractors for the model.

3. By carefully tuning the parameters, we found that the variables in Fig. 1 have more effective impact on the properties of attractors. Specifically, by tuning the degradation rate, activation rate and interaction intensity of the important variables in Fig. 1, we identified the parameter space that can generate tristability. The parameter values are shown in Table S1 (column "Value used in this work").

4. We also tuned the parameter values to satisfy some biological constrains. For example, the three stable states should match the gene expression for G0, G1, S state, relatively.

### Landscape in different coordinates

Landscape is displayed using different coordinates (Fig. S2A-D). The blue region on the landscape represents lower potential or higher probability while the yellow region represents higher potential or lower probability. The landscape results are supported by the single-cell data [2] (Fig. S3A-B). The CycA axis is the relative concentration of CycA. The phospho-Rb axis denotes the phosphorylation level of Rb (Fig. S3B), which decreases as pRb increases. On the landscape, the S state is the lowest basin describing the highest occupation probability of cell states (Fig. S3A), which happens to match the S cluster with the largest number of cells from single cell data (Fig. S3B).

To support three clusters in singel-cell data, we showed the single cell data for the concentration of p21 and the amount of DNA (Fig. S4A). The DNA axis denotes the amount of DNA, and the p21

axis denotes the p21 concentration. We also showed the single cell data for the phosphorylation level of Rb and the amount of DNA (Fig. S4B). The phospho-Rb axis denotes the phosphorylation level of Rb, including the hypo- and hyper- phosphorylated Rb, and DNA axis denotes the amount of DNA. Each point corresponds to a single cell. The G1 cluster and S cluster are separated well in Fig. S4, which supports that there are three attractors in proliferation-quiescence decision system.

We further employed gap statistic method to identify the optimal clustering number [3] (Fig. S5). Gap statistic value can be viewed as the difference between the loss of the random sample and the loss of the actual sample. Therefore, higher scores correspond to better clustering. The horizontal axis represents the number of clusters. The vertical axis represents the gap statistic value. We denoted the optimal number of clusters with blue dot and gray line. Fig. S5A is the result of pRb vs p21, which corresponds to data in Fig. 1D. Fig. S5B is the result of pRb vs CycA, which corresponds to data in Fig. S3B. Fig. S5C is the result of pRb vs DNA, which corresponds to data in Fig. S4A. Fig. S5D is the result of p21 vs DNA, which corresponds to data in Fig. S4B. The highest score in all the subfigures of Fig. S5 is when the number of clusters is three, which supports the three clusters in single cell data and the tristble landscape in our model.

## Landscape changes with the perturbations of key regulators

To study how different factors influence the landscape, we make perturbations to key regulators, individually (Fig. S6-S10). The total level of E2F is controlled by the synthesis rate of E2F (Fig. S6). The cells with low level of E2F are trapped in G0 state (Fig. S6A). As the E2F synthesis rate increases, S and G1 phase will occur (Fig. S6B and C) and the landscape will display a tristable state (Fig. S6D). G0 state disappears after E2F goes above a certain threshold (Fig. S6E). The disappearance of G0 state suggests that the function of RP cannot be maintained at high E2F concentration. Finally, as E2F continues to increase, only a stable S basin is left (Fig. S6F).

Low expression level of Emi1 is not enough to maintain the proliferation (Fig. S7A). Middle level of Emi1 keeps the landscape in tristable state (Fig. S7B and C). However, the landscape with high level of Emi1 suggests that the overexpression of Emi1 may prevent the proliferation (Fig. S7D). Although high level of Cdh1 eliminates the S state (Fig. S8A), extremely low level of Cdh1 will not eliminate any states (Fig. S7E), which corresponds to the observation in previous experiments [4]. The patterns of CycE are similar with CycA (Fig. S8 and S9). The cells with low level of CycE and CycA will undergo a G1 arrest

(Fig. S8A and S9A).

## The role of CycA in G0 to G1 and G1 to S transition

CycA will slightly increase both the G0->G1 action and the G1->G0 action (Fig. S11A). The G0->G1 barrier and the G1->G0 barrier increase as CycA increases (Fig. S11B). Moreover, both the action and barrier ratio between G0->G1 and G1->G0 almost keep constant (Fig. S11C). These results suggest that CycA cannot sustain Rb hyperphosphorylation in the RP [5].

The G1->S transition action is higher than the S->G1 action when CycA is at an extremely low concentration (Fig. S12A), implying that the G1->S transition is more difficult than the S->G1 transition with low concentration of CycA. When the concentration of CycA is high, the G1->S action is lower than the S->G1 action, indicating that the G1->S transition is easier than the S->G1 transition with high concentration of CycA. The S->G1 barrier is higher than the G1->S barrier (Fig. S12B). Both the ratio of action and the ratio of barrier between the G1->S transition and the S->G1 transition decline with CycA accumulation (Fig. S12C). These results reflect that high level of CycA aids the G1->S more than the S->G1 transition, which is supported by previous experiments [6]. On the other hand, the elevation of CycA promotes the absolute value of both G1->S action and S->G1 action, which means that extremely high level of CycA might prevent the cell cycle progression [7].

## The role of CycE in G0 to G1 and G1 to S transition

CycE makes the G0->G1 action and the G1->G0 action (Fig. S13A) increasing. Both the G0->G1 barrier and the G1->G0 barrier are promoted by CycE (Fig. S13B). The action and barrier ratio between G0->G1 and G1->G0 have no significant change (Fig. S13C). These results indicate that CycE has no significant positive impact on the the RP, which is different with the formal hypothesis that CycE should hyperphosphorylate the pRb and facilitate the G0->G1 transition [8,9]. The performance of CycE suggests that CycE is not enough to maintain the Rb hyperphosphorylation in the RP, which is supported by recent research [5]. They reported that CycE and CycA can sustain Rb hyperphosphorylation only from the start of the S phase while CycD performs this job in the RP. Their experimental results also propose that the RP is rather a probabilistic event than an irreversible progress, which agree with our landscape model.

When it comes to G1/S checkpoint, CycE promotes the S->G1 action and reduces the G1->S action

(Fig. S14A). The S->G1 barrier and the G1->S barrier decline with the acceleration of CycE synthesis (Fig. S14B). CycE decreases the action and barrier ratio between G1->S and S->G1 (Fig. S14C). These patterns prove that CycE facilitates the G1->S transition and inhibits the S->G1 transition [6].

## Global sensitivity analysis

We listed the rest of global sensitivity analysis results here including G0 to G1, G1 to S and G0 to S (Fig. S15-S19). Fig. S15 is the result of parameters for the synthetic rates of proteins or transcription factors. Fig. S16 is the result of parameters for the degradation rates. Fig. S17-S19 are the results of parameters for the intensity of regulations. We altered each activated or inhibitory parameters respectively to see how the transition actions between G0 and G1 (Fig. S17), between G1 and S (Fig. S18), as well as between G0 and S (Fig. S15, S16, S19) are influenced. We increased (Subfigure A for Fig. S15-S19) and decreased (Subfigure B for Fig. S15-S19) each of the 45 parameters by 5%, respectively.

The $\Delta S/S$ axis represents the ratio between the change of transition action and the transition action with default parameter values. Magenta bars represent the transition reversing the cell cycle, and cyan bars represent the transition for cell cycle progression. Here, "Sy" is the abbreviation of "Synthesis", i.e. "kSyE2F" means the synthesis rate of E2F. "De" means "Degradation", i.e. "kDeCa" means the degradation rate of CycA. "As" means "Assembly", i.e. "AsRbE2F" means the assembly rate of Rb and E2F. "Ds" means "Disassembly", i.e. "DsRbE2F" means the disassembly rate of the Rb with E2F. "Ph" means "phosphorylation", i.e. "PhC1" means the phosphorylation rate of Cdh1. "Dp" means "dephosphorylation", i.e. "DpRc" means dephosphorylation rate of replication complex. Of note, the sensitivity analysis which we performed here is based on the transition actions between the attractors, which are directly calculated from the multi-dimensional system.

## Network structure perturbation

We randomly removed and reassigned 20 edges individually to study the influence of the network structure changes on the global stability of the system (Table S2 and Table S3). Here, we focus on the change of the number of stable states and the energy landscape shape. "Edge" column represents the edge that we deleted or reassigned. "Num" column represents the number of steady states in the system. "1" under "G0", "G1", and "S" column represents the existence of the corresponding steady state, while "0" represents there is no such state. Due to the large number of model variables, we list the values of seven

important variables when they reach their steady states.

To visualize the effect of network structure perturbation on the system stability, we showed some instances of landscape change for link deletion and link reassignment perturbations, individually (Fig. S20 and Fig. S21). In Fig. S20, random edge deletions cause landscape alteration. Deleting the inhibitory effect of E2F on Rb leads to a "G0" only state (Table S2 and Fig. S20A). Deleting the inhibitory effect of p53 on DNA damage leads to "G0" and "G1" bistable state (Table S2 and Fig. S20B). Deleting the inhibitory effect of Cdh1 on CycA leads to three stable states (Table S2 and Fig. S20C). In Fig. S21, random edge reassignments also cause landscape alteration. Changing the inhibitory regulation of Rb on E2F to the inhibitory regulation of p21 on E2F leads to a "G0" only state (Table. S3 and Fig. S21A). Changing the inhibitory regulation of CycE on p21 to the inhibitory regulation of CycE on Emi1 leads to "G0" and "G1" state (Table S3 and Fig. S21B). Changing the inhibitory regulation of CycA on CycE to the inhibitory regulation of CycA on E2F leads to three stable states (Table S3 and Fig. S21C).

# Supporting tables

**Table S1.** Stable steady states for tristable system.

| Variables | G0 Value | G1 Value | G1 Value | Unit |
|---|---|---|---|---|
| Rb | 0.278595519 | 0.061135886 | 0.016154799 | AU |
| E2Ft | 1.541888198 | 1.990132998 | 2.301559196 | AU |
| E2F | 0.054746835 | 0.285960793 | 1.115495193 | AU |
| E1t | 0.000087010 | 0.000609202 | 1.946859331 | AU |
| CycE | 0.000024377 | 0.000144077 | 0.199390623 | AU |
| CycA | 0.000026867 | 0.000163033 | 0.294415378 | AU |
| C1 | 0.944754735 | 0.711065117 | 0.002272405 | AU |
| E1C1 | 0.054746835 | 0.285960793 | 0.142065528 | AU |
| P21 | 1.883136048 | 1.881211094 | 0.633068987 | AU |
| CeP21 | 0.036468610 | 0.190341174 | 0.101434752 | AU |
| CaP21 | 0.000126847 | 0.000901901 | 0.114617663 | AU |
| RC | 1 | 1 | 0 | AU |
| RCp | 0 | 0 | 0 | AU |
| RCa | 0 | 0 | 0 | AU |
| RCi | 0 | 0 | 0 | AU |
| DNA | 0 | 0 | 1 | AU |
| $Pcna_a$ | 0.000068683 | 0.000077728 | 0.084141818 | AU |
| $Pcna_i$ | 0.499931316 | 0.499922271 | 0.415858181 | AU |
| DAM | 0.328600641 | 0.328600641 | 0.328600641 | AU |
| P53 | 0.338600641 | 0.338600641 | 0.338600641 | AU |

RC pre-replication complexes; $RC_a$ active form of replication complexes
$RC_I$ inactive form of replication complexes;$RC_p$ primed replication complexes

**Table S2.** Random edge deletion.

| Edge | Num | G0 | G1 | S | Rb | | | E2F | | | Emi1 | | | CycE | | | CycA | | | Cdh1 | | | p21 | | |
|---|---|---|---|---|---|---|---|---|---|---|---|---|---|---|---|---|---|---|---|---|---|---|---|---|---|
| | | | | | G0 | G1 | S | G0 | G1 | S | G0 | G1 | S | G0 | G1 | S | G0 | G1 | S | G0 | G1 | S | G0 | G1 | S |
| CycA−\|Rb | 2 | 1 | 1 | 0 | 0.28 | 6.1E-2 | / | 5.4E-2 | 0.28 | / | 8.7E-5 | 6.0E-4 | / | 2.4E-5 | 1.4E-4 | / | 2.7E-5 | 1.6E-4 | / | 0.94 | 0.71 | / | 1.88 | 1.87 | / |
| CycD−\|Rb | 1 | 1 | 0 | 0 | 4.29 | / | / | 9.2E-4 | / | / | 1.4E-6 | / | / | 4.0E-7 | / | / | 4.4E-7 | / | / | 1.00 | / | / | 1.88 | / | / |
| CycE−\|Rb | 2 | 1 | 1 | 0 | 0.29 | 7.1E-2 | / | 5.4E-2 | 0.28 | / | 8.7E-5 | 6.0E-4 | / | 2.5E-5 | 1.4E-4 | / | 2.6E-5 | 1.6E-4 | / | 0.94 | 0.71 | / | 1.88 | 1.87 | / |
| E2F−\|Rb | 1 | 1 | 0 | 0 | 1.73 | / | / | 7.8E-3 | / | / | 1.2E-5 | / | / | 3.4E-6 | / | / | 3.7E-6 | / | / | 0.99 | / | / | 1.88 | / | / |
| Rb−\|E2F | 1 | 0 | 0 | 1 | / | / | 7.7E-3 | / | / | 2.39 | / | / | 4.68 | / | / | 0.26 | / | / | 0.99 | / | / | 1.0E-3 | / | / | 0.31 |
| p21−\|CycE | 1 | 0 | 0 | 1 | / | / | 1.5E-2 | / | / | 1.30 | / | / | 2.39 | / | / | 0.31 | / | / | 0.42 | / | / | 1.9E-3 | / | / | 0.48 |
| p21−\|CycA | 2 | 1 | 0 | 1 | 0.28 | / | 1.4E-2 | 5.5E-2 | / | 1.31 | 8.8E-5 | / | 2.46 | 2.4E-5 | / | 0.20 | 1.6E-4 | / | 0.56 | 0.94 | / | 1.9E-3 | 1.88 | / | 0.47 |
| E2F− >E2F | 1 | 1 | 0 | 0 | 0.51 | / | / | 2.5E-2 | / | / | 3.9E-5 | / | / | 1.1E-5 | / | / | 1.2E-5 | / | / | 0.97 | / | / | 1.88 | / | / |
| Cdh1−\|CycA | 3 | 1 | 1 | 1 | 0.28 | 6.1E-2 | 1.3E-2 | 5.5E-2 | 0.29 | 1.34 | 8.7E-5 | 6.2E-4 | 2.53 | 2.5E-5 | 1.7E-4 | 0.20 | 4.0E-5 | 2.7E-4 | 0.61 | 0.94 | 0.70 | 1.8E-3 | 1.88 | 1.87 | 0.45 |
| CycA−\|CycE | 3 | 1 | 1 | 1 | 0.28 | 6.1E-2 | 1.1E-2 | 5.5E-2 | 0.29 | 1.71 | 8.7E-5 | 6.1E-4 | 3.28 | 2.4E-5 | 1.4E-4 | 1.07 | 2.7E-5 | 1.6E-4 | 0.68 | 0.94 | 0.71 | 1.4E-3 | 1.88 | 1.87 | 0.23 |
| Cdh1−\|Emi1 | 3 | 1 | 1 | 1 | 0.28 | 6.1E-2 | 1.6E-2 | 5.5E-2 | 0.29 | 1.16 | 0.11 | 0.57 | 2.31 | 2.5E-5 | 1.6E-4 | 0.21 | 3.5E-5 | 2.4E-4 | 0.33 | 9.1E-3 | 1.8E-3 | 1.9E-3 | 1.88 | 1.87 | 0.59 |
| CycE−\|p21 | 2 | 1 | 1 | 0 | 0.28 | 6.1E-2 | / | 5.5E-2 | 0.29 | / | 8.7E-5 | 6.1E-4 | / | 2.3E-5 | 1.4E-4 | / | 2.6E-5 | 1.6E-4 | / | 0.94 | 0.71 | / | 1.90 | 1.89 | / |
| CycA−\|p21 | 2 | 1 | 1 | 0 | 0.28 | 6.1E-2 | / | 5.5E-2 | 0.29 | / | 8.7E-5 | 6.1E-4 | / | 2.4E-5 | 1.5E-4 | / | 2.7E-5 | 1.7E-4 | / | 0.94 | 0.71 | / | 1.89 | 1.88 | / |
| aRC−\|p21 | 3 | 1 | 1 | 1 | 0.28 | 6.1E-2 | 1.6E-2 | 5.5E-2 | 0.28 | 1.12 | 8.7E-5 | 6.1E-4 | 1.96 | 2.4E-5 | 1.4E-4 | 0.20 | 2.7E-5 | 1.6E-4 | 0.30 | 0.94 | 0.71 | 2.3E-3 | 1.88 | 1.87 | 0.65 |
| CycA−\|Cdh1 | 2 | 1 | 1 | 0 | 0.28 | 6.1E-2 | / | 5.5E-2 | 0.29 | / | 8.7E-5 | 6.0E-4 | / | 2.4E-5 | 1.4E-4 | / | 2.7E-5 | 1.6E-4 | / | 0.95 | 0.71 | / | 1.88 | 1.87 | / |
| CycE−\|Cdh1 | 3 | 1 | 1 | 1 | 0.28 | 6.1E-2 | 1.6E-2 | 5.5E-2 | 0.29 | 1.12 | 8.7E-5 | 6.1E-4 | 1.94 | 2.4E-5 | 1.4E-4 | 0.20 | 2.7E-5 | 1.6E-4 | 0.29 | 0.95 | 0.71 | 2.5E-3 | 1.88 | 1.87 | 0.63 |
| p53− >p21 | 3 | 1 | 1 | 1 | 0.28 | 5.7E-2 | 1.4E-2 | 5.5E-2 | 0.31 | 1.29 | 8.8E-5 | 7.1E-4 | 2.39 | 1.2E-4 | 2.0E-3 | 0.24 | 8.0E-5 | 8.3E-4 | 0.48 | 0.94 | 0.68 | 1.9E-3 | 0.80 | 0.79 | 0.21 |
| aRC− >pcna | 3 | 1 | 1 | 1 | 0.28 | 6.1E-2 | 1.6E-2 | 5.5E-2 | 0.29 | 1.11 | 8.7E-5 | 6.1E-4 | 1.95 | 2.4E-5 | 1.4E-4 | 0.20 | 2.7E-5 | 1.6E-4 | 0.29 | 0.94 | 0.71 | 2.2E-3 | 1.88 | 1.87 | 0.63 |
| aRC− >DAM | 3 | 1 | 1 | 1 | 0.28 | 6.1E-2 | 1.6E-2 | 5.5E-2 | 0.29 | 1.12 | 8.7E-5 | 6.1E-4 | 1.95 | 2.4E-5 | 1.4E-4 | 0.20 | 2.7E-5 | 1.6E-4 | 0.29 | 0.94 | 0.71 | 2.2E-3 | 1.88 | 1.87 | 0.63 |
| p53−\|DAM | 2 | 1 | 1 | 0 | 0.28 | 6.1E-2 | / | 5.5E-2 | 0.28 | / | 8.7E-5 | 6.0E-4 | / | 9.4E-6 | 5.1E-5 | / | 1.2E-5 | 6.4E-5 | / | 0.95 | 0.71 | / | 4.0 | 4.0 | / |

**Table S3.** Random edge reassignment.

<table>
<tr><th rowspan="2">Edge</th><th rowspan="2">Num</th><th rowspan="2">G0</th><th rowspan="2">G1</th><th rowspan="2">S</th><th colspan="3">Rb</th><th colspan="3">E2F</th><th colspan="3">Emi1</th><th colspan="3">CycE</th><th colspan="3">CycA</th><th colspan="3">Cdh1</th><th colspan="3">p21</th></tr>
<tr><th>G0</th><th>G1</th><th>S</th><th>G0</th><th>G1</th><th>S</th><th>G0</th><th>G1</th><th>S</th><th>G0</th><th>G1</th><th>S</th><th>G0</th><th>G1</th><th>S</th><th>G0</th><th>G1</th><th>S</th><th>G0</th><th>G1</th><th>S</th></tr>
<tr><td>CycE−|p21 change to CycE−| Emi1</td><td>2</td><td>1</td><td>1</td><td>0</td><td>0.28</td><td>6.1E-2</td><td>/</td><td>5.5E-2</td><td>0.29</td><td>/</td><td>0</td><td>0</td><td>/</td><td>2.4E-5</td><td>1.4E-4</td><td>/</td><td>2.5E-5</td><td>1.5E-4</td><td>/</td><td>1.44</td><td>1.21</td><td>/</td><td>1.89</td><td>1.88</td><td>/</td></tr>
<tr><td>CycA−|p21 change to CycA−| Emi1</td><td>2</td><td>1</td><td>1</td><td>0</td><td>0.28</td><td>6.1E-2</td><td>/</td><td>5.5E-2</td><td>0.29</td><td>/</td><td>0</td><td>0</td><td>/</td><td>2.4E-5</td><td>1.4E-4</td><td>/</td><td>2.5E-5</td><td>1.5E-4</td><td>/</td><td>1.44</td><td>1.21</td><td>/</td><td>1.88</td><td>1.87</td><td>/</td></tr>
<tr><td>Rb−|E2F change to Rb−|p21</td><td>1</td><td>0</td><td>0</td><td>1</td><td>/</td><td>/</td><td>7.6E-3</td><td>/</td><td>/</td><td>2.39</td><td>/</td><td>/</td><td>4.76</td><td>/</td><td>/</td><td>9.2E-2</td><td>/</td><td>/</td><td>4.58</td><td>/</td><td>/</td><td>9.5E-4</td><td>/</td><td>/</td><td>6.6E-6</td></tr>
<tr><td>Rb−|E2F change to Rb− >p53</td><td>1</td><td>0</td><td>0</td><td>1</td><td>/</td><td>/</td><td>7.7E-3</td><td>/</td><td>/</td><td>2.39</td><td>/</td><td>/</td><td>4.69</td><td>/</td><td>/</td><td>0.25</td><td>/</td><td>/</td><td>1.04</td><td>/</td><td>/</td><td>1.0E-3</td><td>/</td><td>/</td><td>0.13</td></tr>
<tr><td>CycA−|Rb change to CycA−|E2F</td><td>2</td><td>1</td><td>1</td><td>0</td><td>0.28</td><td>6.2E-2</td><td>/</td><td>5.5E-2</td><td>0.28</td><td>/</td><td>8.7E-5</td><td>6.0E-4</td><td>/</td><td>2.4E-5</td><td>1.4E-4</td><td>/</td><td>2.7E-5</td><td>1.5E-4</td><td>/</td><td>0.94</td><td>0.71</td><td>/</td><td>1.88</td><td>1.87</td><td>/</td></tr>
<tr><td>CycE−|Rb change to CycE−|E2F</td><td>2</td><td>1</td><td>1</td><td>0</td><td>0.28</td><td>6.2E-2</td><td>/</td><td>5.5E-2</td><td>0.28</td><td>/</td><td>8.7E-5</td><td>6.0E-4</td><td>/</td><td>2.4E-5</td><td>1.4E-4</td><td>/</td><td>2.7E-5</td><td>1.6E-4</td><td>/</td><td>0.94</td><td>0.71</td><td>/</td><td>1.88</td><td>1.87</td><td>/</td></tr>
<tr><td>CycE−|Rb change to CycE− >Rb</td><td>2</td><td>1</td><td>1</td><td>0</td><td>0.28</td><td>6.2E-2</td><td>/</td><td>5.5E-2</td><td>0.28</td><td>/</td><td>8.7E-5</td><td>6.0E-4</td><td>/</td><td>2.4E-5</td><td>1.4E-4</td><td>/</td><td>2.7E-5</td><td>1.6E-4</td><td>/</td><td>0.94</td><td>0.71</td><td>/</td><td>1.88</td><td>1.87</td><td>/</td></tr>
<tr><td>CycA−|Rb change to CycA− >Rb</td><td>2</td><td>1</td><td>1</td><td>0</td><td>0.28</td><td>6.2E-2</td><td>/</td><td>5.5E-2</td><td>0.28</td><td>/</td><td>8.7E-5</td><td>6.0E-4</td><td>/</td><td>2.4E-5</td><td>1.4E-4</td><td>/</td><td>2.7E-5</td><td>1.6E-4</td><td>/</td><td>0.94</td><td>0.71</td><td>/</td><td>1.88</td><td>1.87</td><td>/</td></tr>
<tr><td>CycD−|Rb change to CycD−|p21</td><td>1</td><td>1</td><td>0</td><td>0</td><td>4.28</td><td>/</td><td>/</td><td>9.2E-4</td><td>/</td><td>/</td><td>1.4E-6</td><td>/</td><td>/</td><td>6.1E-4</td><td>/</td><td>/</td><td>2.4E-6</td><td>/</td><td>/</td><td>1.00</td><td>/</td><td>/</td><td>3.6E-2</td><td>/</td><td>/</td></tr>
<tr><td>p53− >p21 change to p53−|p21</td><td>1</td><td>0</td><td>0</td><td>1</td><td>/</td><td>/</td><td>1.3E-2</td><td>/</td><td>/</td><td>1.32</td><td>/</td><td>/</td><td>2.46</td><td>/</td><td>/</td><td>0.25</td><td>/</td><td>/</td><td>0.52</td><td>/</td><td>/</td><td>1.9E-3</td><td>/</td><td>/</td><td>6.9E-6</td></tr>
<tr><td>pcna−|p21 change to pcna− >p21</td><td>2</td><td>1</td><td>1</td><td>0</td><td>0.28</td><td>6.1E-2</td><td>/</td><td>5.5E-2</td><td>0.29</td><td>/</td><td>8.7E-5</td><td>6.1E-4</td><td>/</td><td>2.4E-5</td><td>1.4E-4</td><td>/</td><td>2.7E-5</td><td>1.6E-4</td><td>/</td><td>0.94</td><td>0.71</td><td>/</td><td>1.88</td><td>1.87</td><td>/</td></tr>
<tr><td>aRC−|p21 change to aRC− >p21</td><td>2</td><td>1</td><td>1</td><td>0</td><td>0.28</td><td>6.1E-2</td><td>/</td><td>5.5E-2</td><td>0.29</td><td>/</td><td>8.7E-5</td><td>6.1E-4</td><td>/</td><td>2.4E-5</td><td>1.4E-4</td><td>/</td><td>2.7E-5</td><td>1.6E-4</td><td>/</td><td>0.94</td><td>0.71</td><td>/</td><td>1.88</td><td>1.87</td><td>/</td></tr>
<tr><td>E2F− >Emi1 change to E2F−|Emi1</td><td>2</td><td>1</td><td>1</td><td>0</td><td>0.28</td><td>6.1E-2</td><td>/</td><td>5.5E-2</td><td>0.29</td><td>/</td><td>0</td><td>0</td><td>/</td><td>2.4E-5</td><td>1.4E-4</td><td>/</td><td>2.6E-5</td><td>1.5E-4</td><td>/</td><td>1.05</td><td>1.28</td><td>/</td><td>1.88</td><td>1.87</td><td>/</td></tr>
<tr><td>CycA−|CycE change to CycA−|CycE</td><td>2</td><td>1</td><td>1</td><td>0</td><td>0.28</td><td>6.1E-2</td><td>/</td><td>5.5E-2</td><td>0.29</td><td>/</td><td>8.7e-5</td><td>6.1E-4</td><td>/</td><td>2.4E-5</td><td>1.4E-4</td><td>/</td><td>2.7E-5</td><td>1.6E-4</td><td>/</td><td>0.94</td><td>0.71</td><td>/</td><td>1.88</td><td>1.87</td><td>/</td></tr>
<tr><td>CycA−|CycE change to CycA−|E2F</td><td>3</td><td>1</td><td>1</td><td>1</td><td>0.28</td><td>6.1E-2</td><td>1.8E-2</td><td>5.5E-2</td><td>0.29</td><td>1.00</td><td>8.7e-5</td><td>6.1E-4</td><td>1.73</td><td>2.4E-5</td><td>1.4E-4</td><td>0.56</td><td>2.7E-5</td><td>1.6E-4</td><td>0.32</td><td>0.94</td><td>0.71</td><td>2.6E-3</td><td>1.88</td><td>1.87</td><td>0.42</td></tr>
<tr><td>p21−|aRC change to p21−|E2F</td><td>1</td><td>1</td><td>0</td><td>0</td><td>1.67</td><td>/</td><td>/</td><td>5.8E-4</td><td>/</td><td>/</td><td>8.7E-7</td><td>/</td><td>/</td><td>4.3E-7</td><td>/</td><td>/</td><td>4.2E-7</td><td>/</td><td>/</td><td>1.00</td><td>/</td><td>/</td><td>1.85</td><td>/</td><td>/</td></tr>
<tr><td>Cdh1−|Emi1 change to Cdh1−|E2F</td><td>2</td><td>1</td><td>1</td><td>0</td><td>0.28</td><td>6.2E-2</td><td>/</td><td>5.5E-2</td><td>0.28</td><td>/</td><td>8.7e-5</td><td>5.9E-4</td><td>/</td><td>2.4E-5</td><td>1.4E-4</td><td>/</td><td>2.7E-5</td><td>1.6E-4</td><td>/</td><td>0.94</td><td>0.72</td><td>/</td><td>1.88</td><td>1.87</td><td>/</td></tr>
<tr><td>Cdh1−|Emi1 change to Cdh1−|Rb</td><td>2</td><td>0</td><td>1</td><td>1</td><td>/</td><td>3.5E-2</td><td>1.0E-2</td><td>/</td><td>0.49</td><td>1.54</td><td>/</td><td>5.15</td><td>21.9</td><td>/</td><td>3.5E-4</td><td>0.21</td><td>/</td><td>5.6E-4</td><td>0.69</td><td>/</td><td>3.0E-4</td><td>2.2E-4</td><td>/</td><td>1.87</td><td>0.41</td></tr>
<tr><td>Cdh1−|CycA change to Cdh1− >CycA</td><td>1</td><td>0</td><td>0</td><td>1</td><td>/</td><td>/</td><td>1.2E-2</td><td>/</td><td>/</td><td>1.55</td><td>/</td><td>/</td><td>3.02</td><td>/</td><td>/</td><td>0.15</td><td>/</td><td>/</td><td>1.10</td><td>/</td><td>/</td><td>1.5E-3</td><td>/</td><td>/</td><td>0.31</td></tr>
<tr><td>Rb−|E2F change to p21−|E2F</td><td>1</td><td>1</td><td>0</td><td>0</td><td>1.03</td><td>/</td><td>/</td><td>7.1E-3</td><td>/</td><td>/</td><td>1.1E-5</td><td>/</td><td>/</td><td>3.1E-6</td><td>/</td><td>/</td><td>3.4E-6</td><td>/</td><td>/</td><td>0.99</td><td>/</td><td>/</td><td>1.88</td><td>/</td><td>/</td></tr>
</table>

# Supporting figures

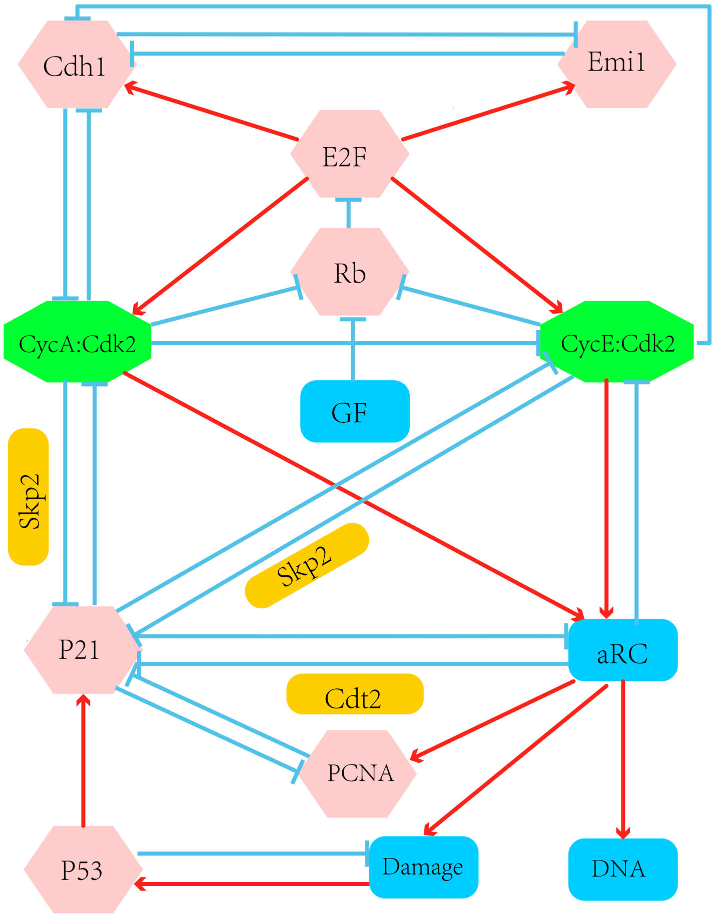


**Figure S1.** A complete influence diagram of the the early cell-cycle model. The red arrows represent activation and the blue bars represent inhibition. Pink hexagons represent transcription factors, green octagons represent protein complexes, and blue quadrilaterals represent cellular components of non-simple structures, including cell damage, cell replication-activated complexes, growth factor and DNA. Yellow rectangles represent the components as parameters.

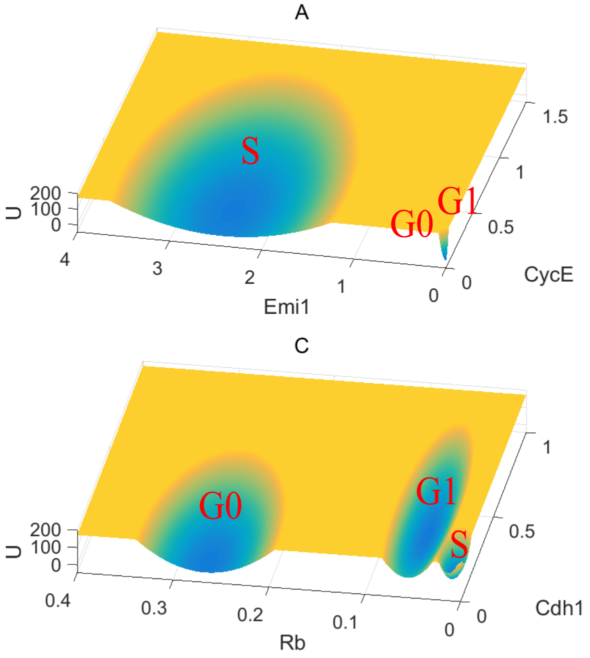


**Figure S2.** The landscape is displayed using different pairs of components. (A) The Emi1-CycE plane. The Emi1 axis is the Emi1 concentration. The CycE axis is the CycE concentration. (B) The P21-E2F plane. The E2F axis is the E2F. The P21 axis is the P21. (C) The Rb-Cdh1 plane. The Rb axis is the pRb concentration. The Cdh1 axis is the Cdh1 concentration. (D) The E2F-Rb plane. The E2F axis is the E2F concentration. The Rb axis is the pRb concentration.

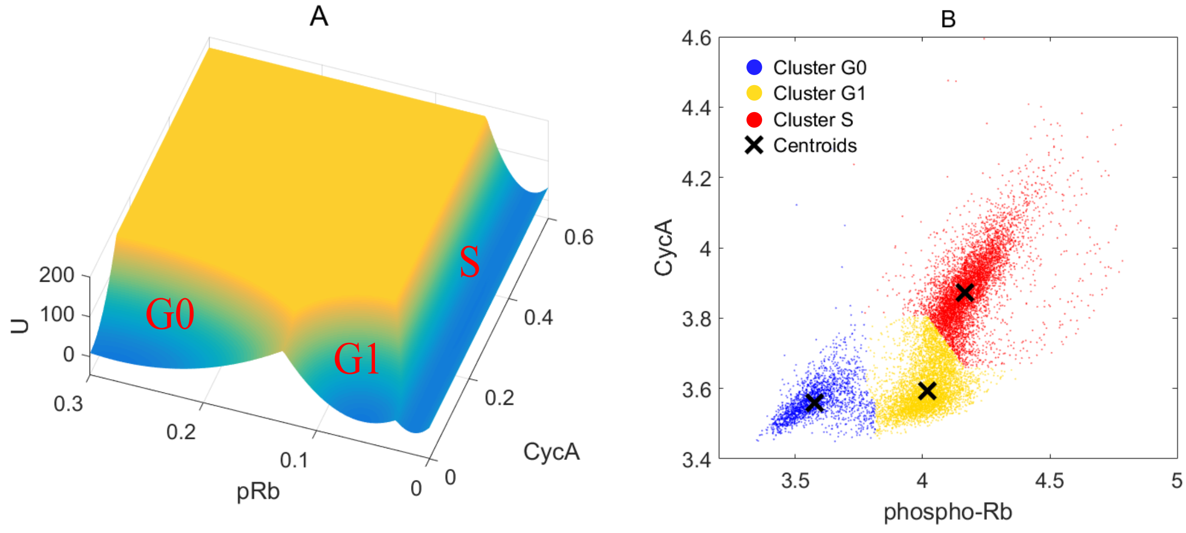


**Figure S3.** Cell quiescence-proliferation fate decision landscape. (A) is the landscape in 3D view. The pRb axis represents the pRb concentration, and the CycA axis represents the CycA concentration (They have different units and we don't annotate them). The U axis represents the potential energy of the points in pRb-CycA plane. The G0 state represents the cells cannot pass the restriction point and enter the G0 quiescence. The G1 state represents the cells cannot pass the G1/S checkpoint and stay in G1 arresting. The S state represents the proliferation state. (B) is the single-cell data of the Rb phosphorylation level versus the CycA concentration. The X axis is the phosphorylation level of Rb, including both the hypo- and hyper-phosphorylated Rb. The Y axis is the CycA concentration. Each point corresponds to a single cell.

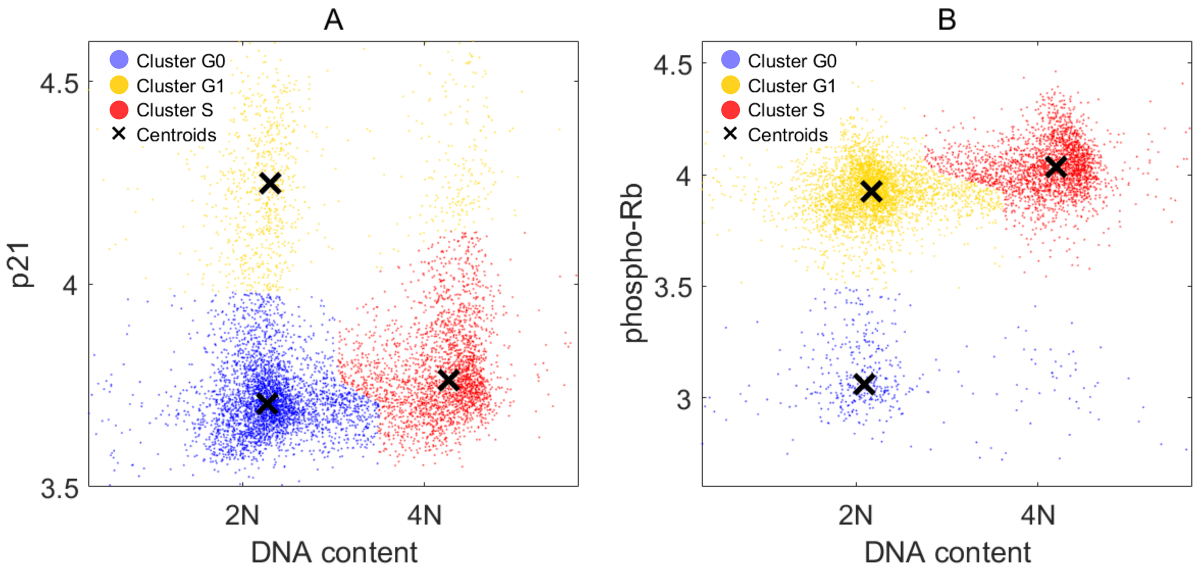


**Figure S4.** Single cell data supporting three stable cell states. (A) The single cell data for the concentration of p21 and the amount of DNA. The DNA axis denotes the amount of DNA. The p21 axis denotes the p21 concentration. Each point corresponds to a single cell. (B) The single cell data for the phosphorylation level of Rb and the amount of DNA. The phospho-Rb axis denotes the phosphorylation level of Rb, including the hypo- and hyper- phosphorylated Rb. The DNA axis denotes the amount of DNA. Each point corresponds to a single cell.

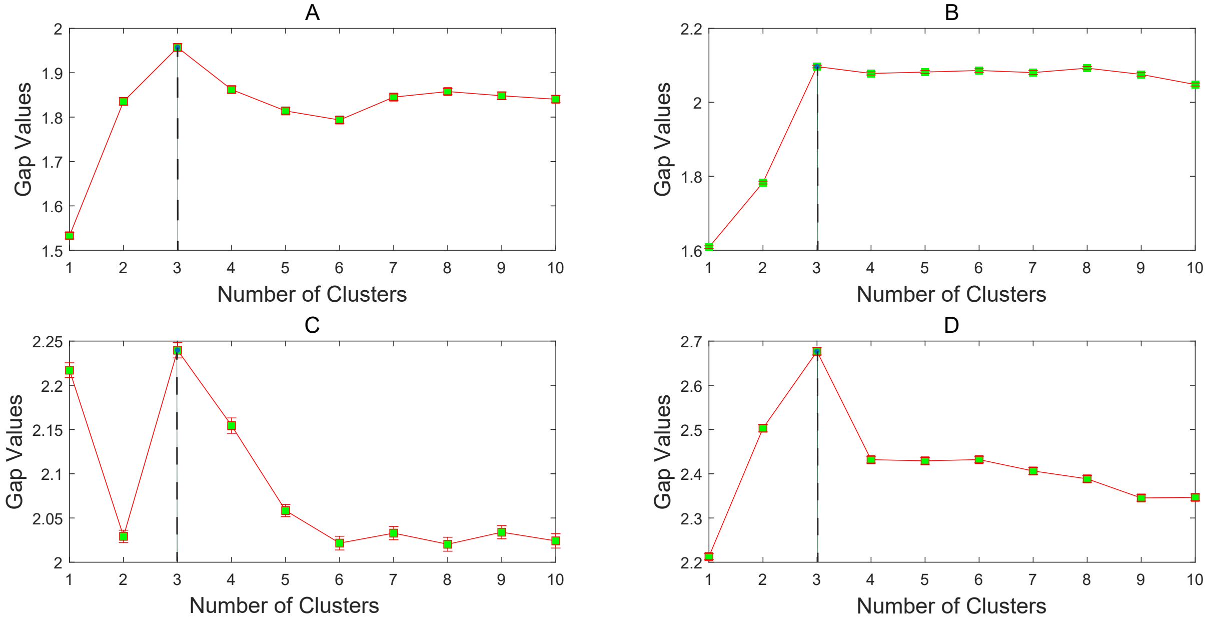


**Figure S5.** Gap statistic results for the single cell data. The horizontal axis represents the number of clusters. The vertical axis represents the gap statistic value. Higher scores correspond to better clustering. Blue dot and the gray line denote the optimal number of clusters. (A) Gap statistic analysis of pRb vs p21, which corresponds to data in Fig. 1D. (B) Gap statistic analysis of pRb vs CycA, which corresponds to data in Fig. S3B. (C) Gap statistic analysis of pRb vs DNA, which corresponds to data in Fig. S4A. (D) Gap statistic analysis of p21 vs DNA, which corresponds to data in Fig. S4B.

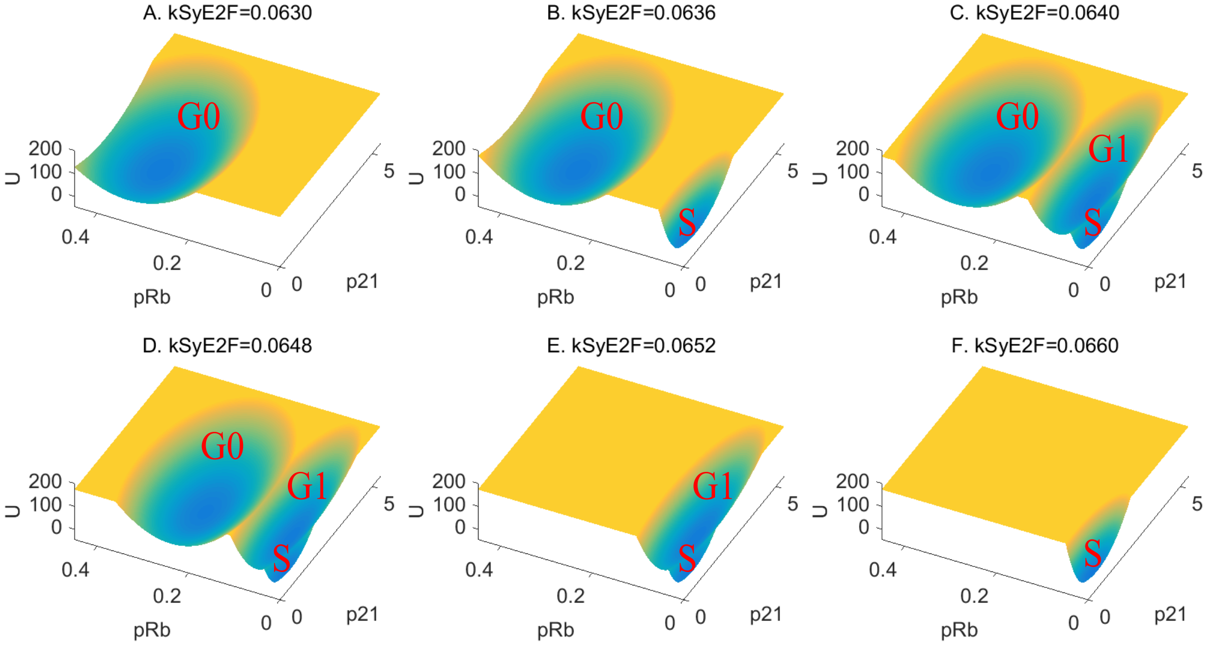


**Figure S6.** Landscape changes as the synthesis rate of E2F changes. kSyE2F is the synthesis rate of E2F. From A to F, the synthesis rate of E2F is increased. The pRb axis represents the pRb concentration, the p21 axis represents the p21 concentration. The U axis is the $-ln(P_{ss})$, $P_{ss}$ represents the steady states probability distribution.

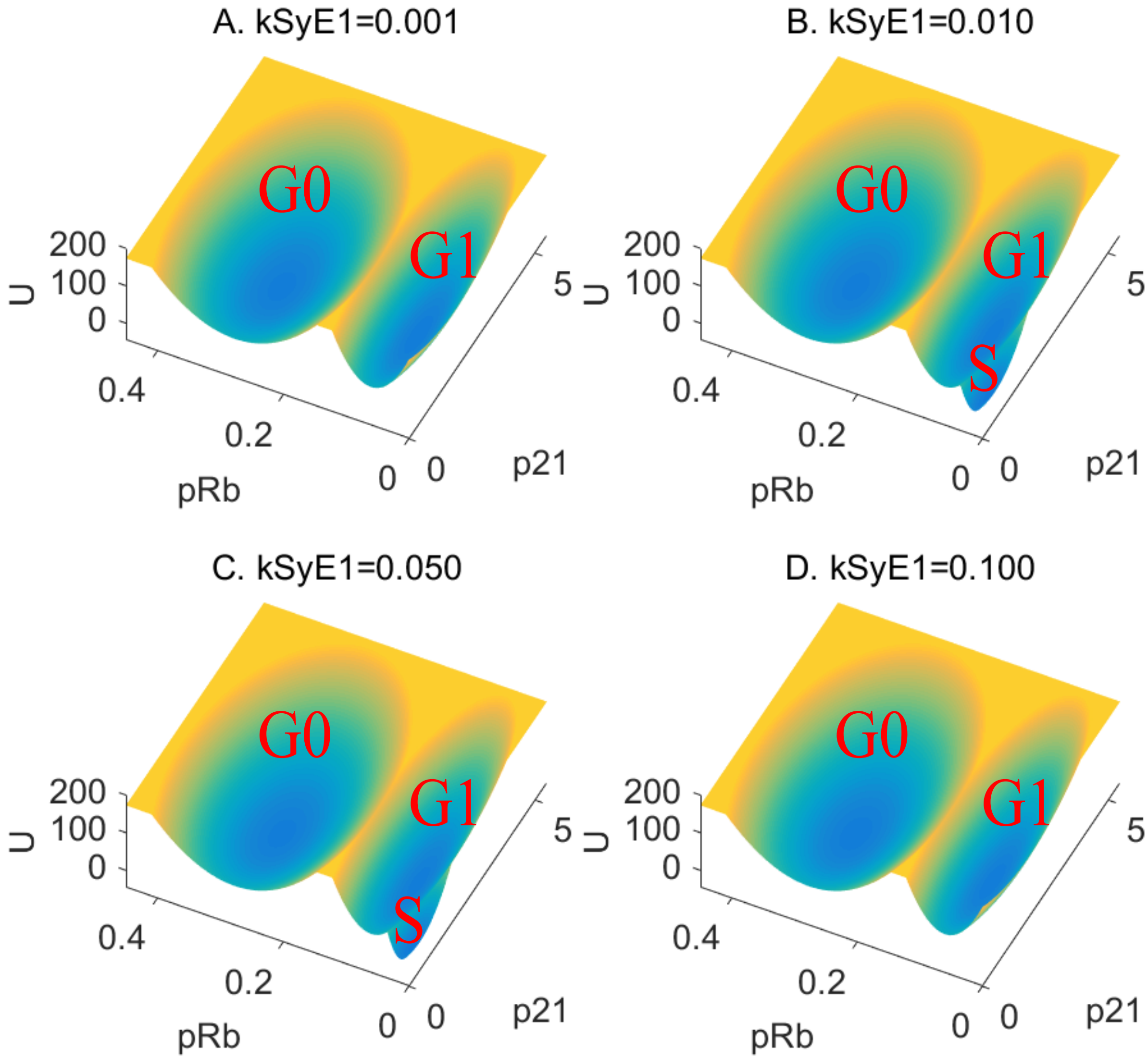


**Figure S7.** Landscape changes as the synthesis rate of Emi1 changes. kSyE1 is the synthesis rate of Emi1. From A to D, the Emi1 synthesis rate is increased. The pRb axis represents the pRb concentration, the p21 axis represents the p21 concentration. The U axis is the $-ln(P_{ss})$, $P_{ss}$ represents the steady states probability distribution.

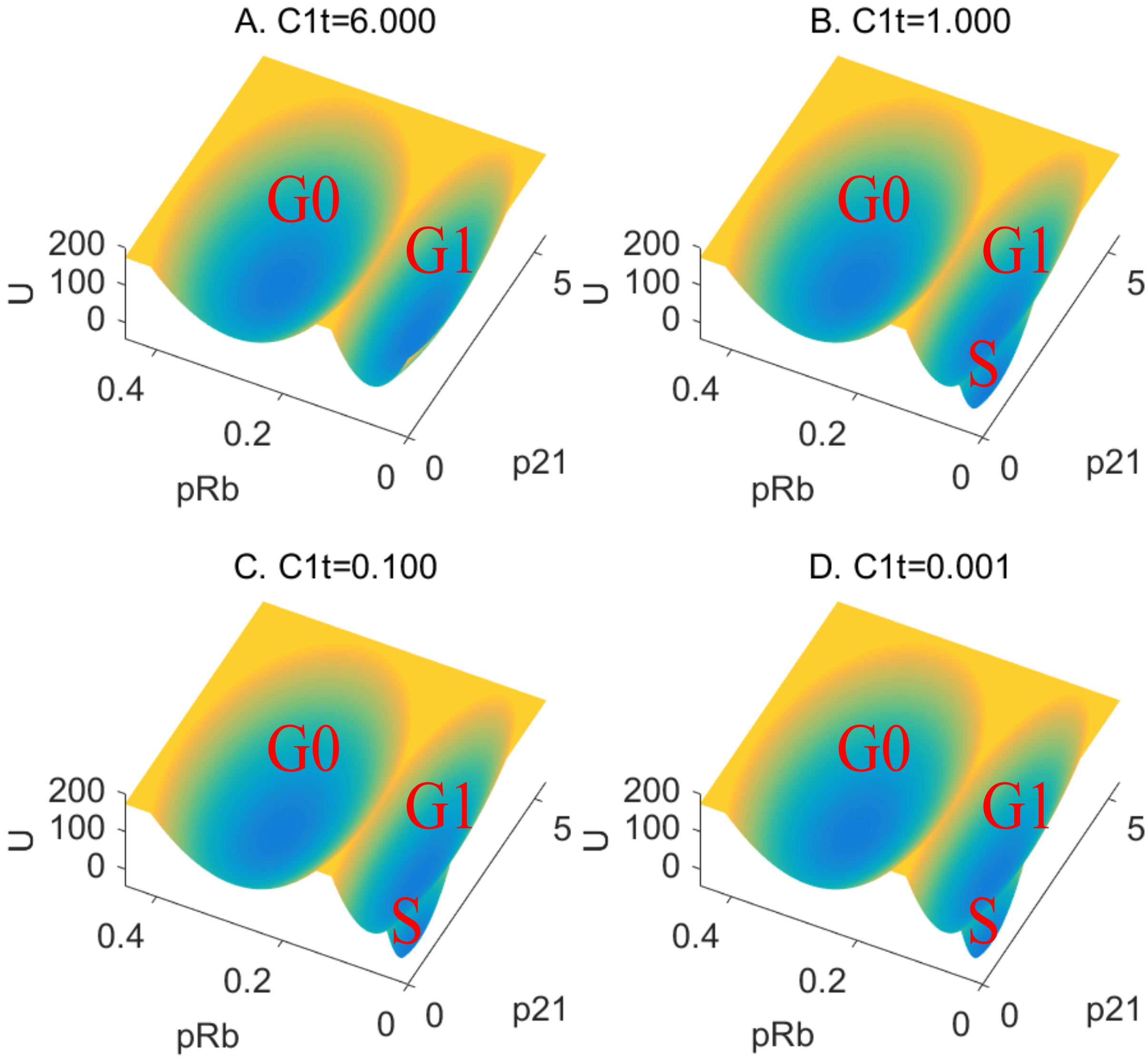


**Figure S8.** Landscape changes as the concentration of Cdh1 changes. C1t is the total concentration of Cdh1. From A to D, the Cdh1 concentration is increased. The pRb axis represents the pRb concentration, the p21 axis represents the p21 concentration. The U axis is the $-ln(P_{ss})$, $P_{ss}$ represents the steady states probability distribution.

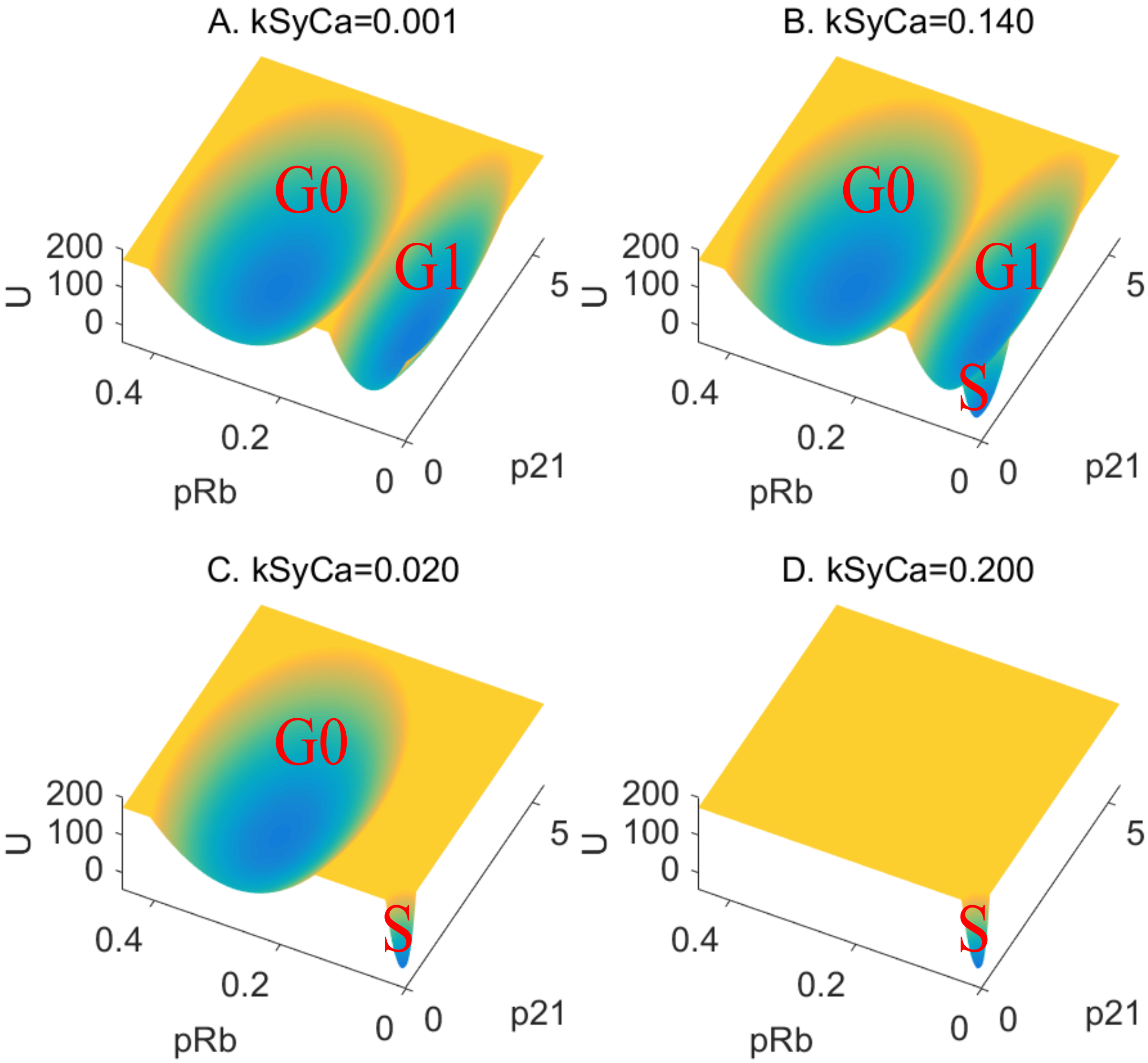


**Figure S9.** Landscape changes as the synthesis rate of CycA changes. kSyCa is the synthesis rate of CycA. From A to D, the CycA synthesis rate is increased. The pRb axis represents the pRb concentration, the p21 axis represents the p21 concentration. The U axis is the $-ln(P_{ss})$, $P_{ss}$ represents the steady states probability distribution.

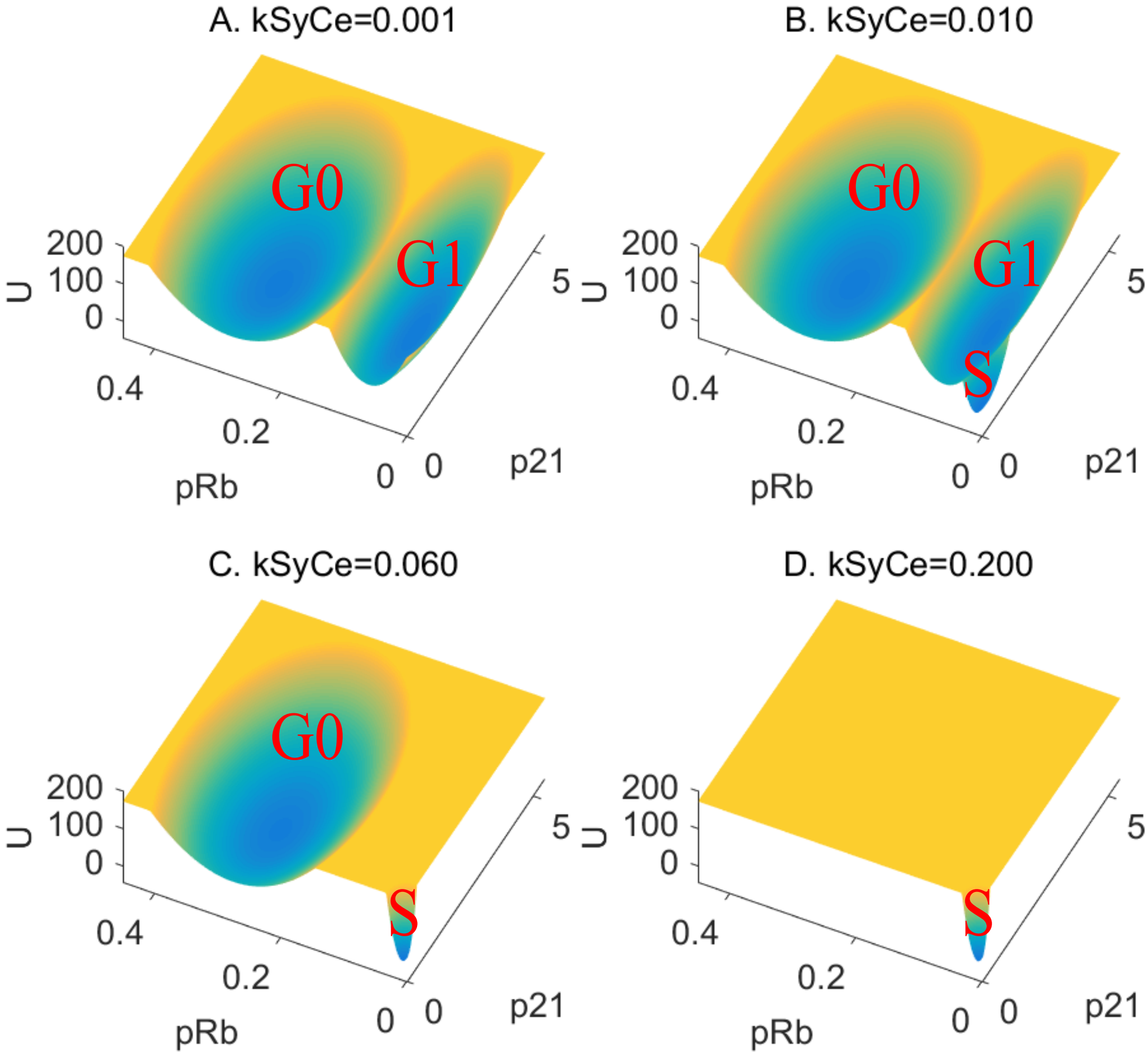


**Figure S10.** Landscape changes as the synthesis rate of CycE changes. kSyCe is the synthesis rate of CycE. From A to D, the synthesis rate of CycE is increased. The pRb axis represents the pRb concentration, the p21 axis represents the p21 concentration. The U axis is the $-ln(P_{ss})$, $P_{ss}$ represents the steady states probability distribution.

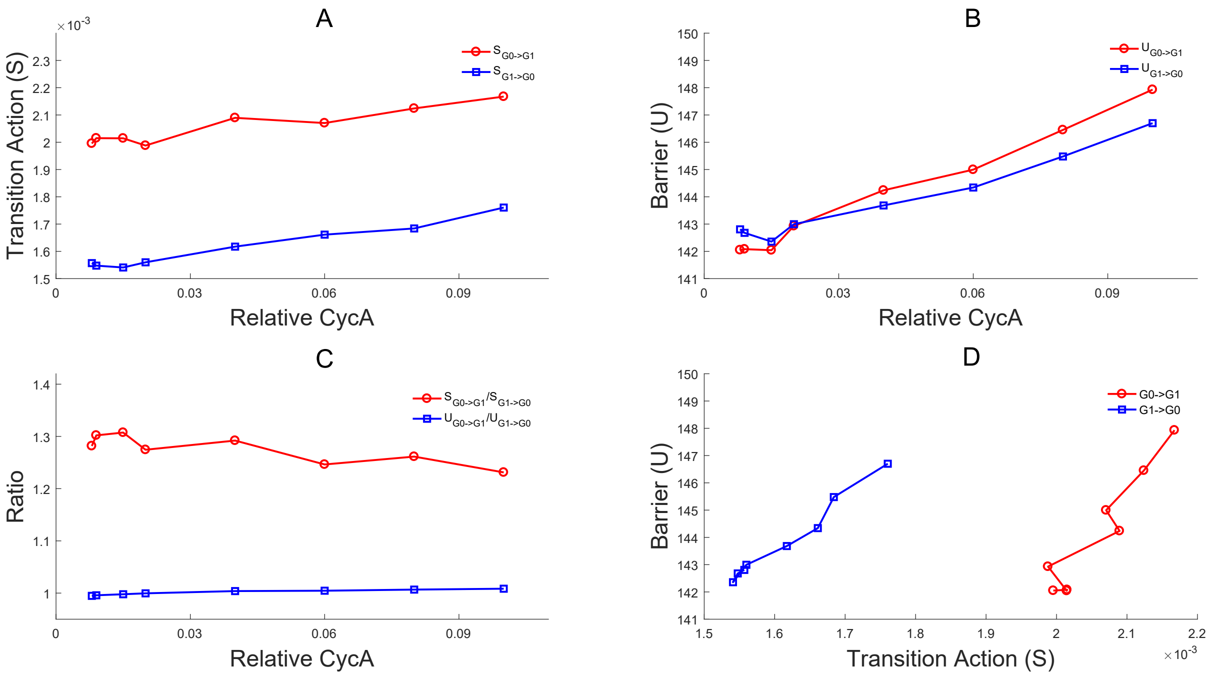


**Figure S11.** Effect of CycA (A-D) on the transition action (S) and barrier (U) between G0 and G1. (A) Effect of CycA on the transition action between G0 and G1. The x axis is the relative concentration of CycA, and the y axis is the value of transition action (S). The red line represents the G0->G1 action and the blue line represents the G1->G0 action. (B) Effect of CycA on barrier (U) between G0 and G1. The x axis is the relative concentration of CycA and the y axis is the barrier (U). The red line represents the G0->G1 barrier, and the blue line represents the G1->G0 barrier. (C) The ratio of the action and the barrier between G0->G1 and G1->G0 changes with CycA. The red line represents the ratio of the action between G0->G1 and G1->G0. The blue line represents the ratio of the barrier between G0->G1 and G1->G0. (D) Transition action (S) versus barrier (U). The red line represents the G0->G1 and the blue line represents G1->G0.

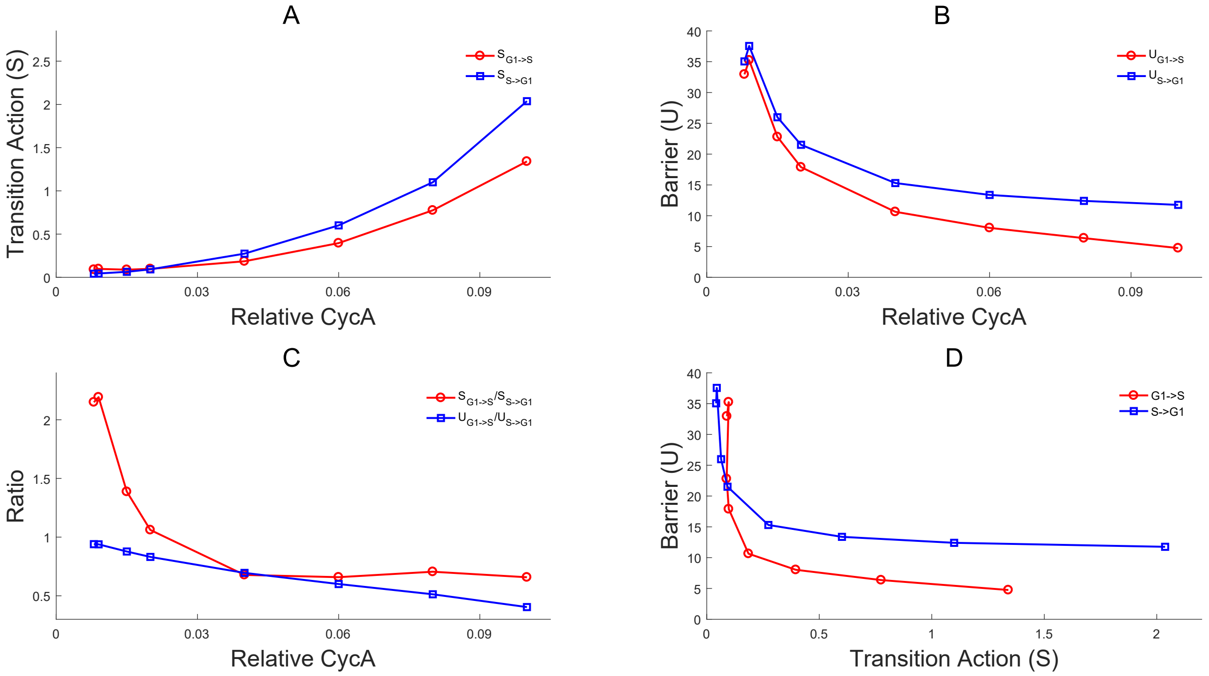


**Figure S12.** Effect of CycA (A-D) on the transition action (S) and barrier (U) between G1 and S. (A) Effect of CycA on the transition action between G1 and S. The x axis is the relative concentration of CycA, and the y axis is the value of transition action (S). The red line represents the G1->S action and the blue line represents the S->G1 action. (B) Effect of CycA on barrier (U) between G1 and S. The x axis is the relative concentration of CycA and the y axis is the barrier (U). The red line represents the G1->S barrier, and the blue line represents the S->G1 barrier. (C) The ratio of the action and the barrier between G1->S and S->G1 changes with CycA. The red line represents the ratio of the action between G1->S and S->G1. The blue line represents the ratio of the barrier between G1->S and S->G1. (D) Transition action (S) versus barrier (U). The red line represents the G1->S and the blue line represents S->G1.

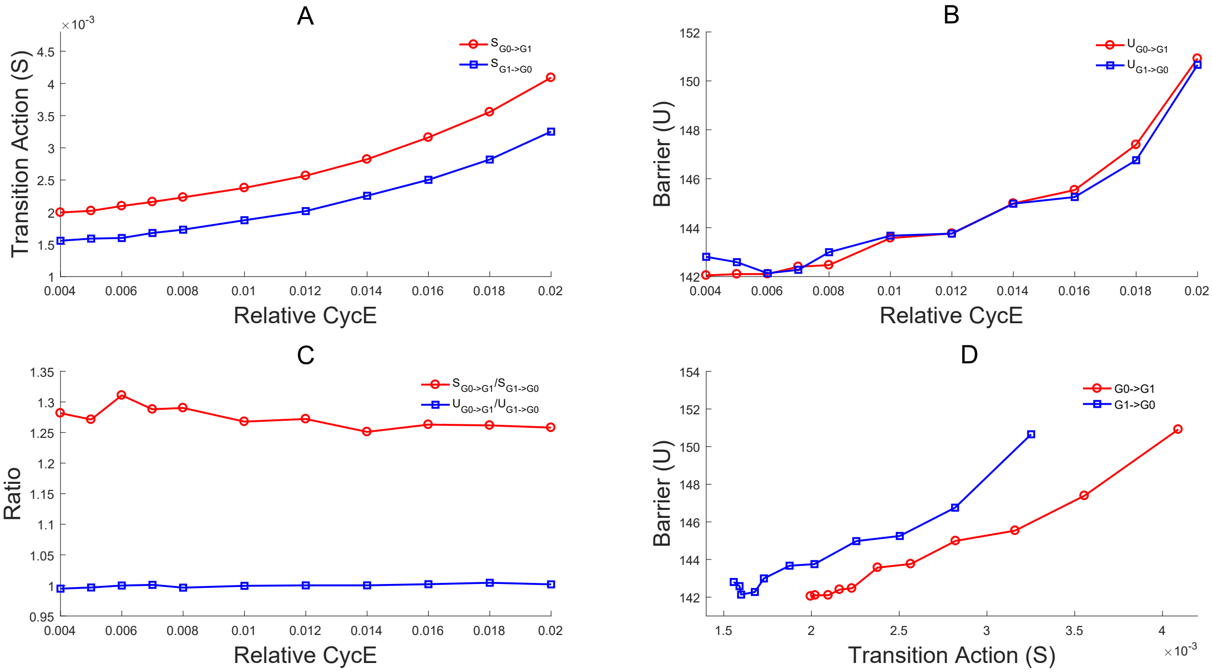


**Figure S13.** Effect of CycE (A-D) on the transition action (S) and barrier (U) between G0 and G1. (A) Effect of CycE on the transition action between G0 and G1. The x axis is the relative concentration of CycE, and the y axis is the value of transition action (S). The red line represents the G0->G1 action and the blue line represents the G1->G0 action. (B) Effect of CycE on barrier (U) between G0 and G1. The x axis is the relative concentration of CycE and the y axis is the barrier (U). The red line represents the G0->G1 barrier, and the blue line represents the G1->G0 barrier. (C) The ratio of the action and the barrier between G0->G1 and G1->G0 changes with CycE. The red line represents the ratio of the action between G0->G1 and G1->G0. The blue line represents the ratio of the barrier between G0->G1 and G1->G0. (D) Transition action (S) versus barrier (U). The red line represents the G0->G1 and the blue line represents G1->G0.

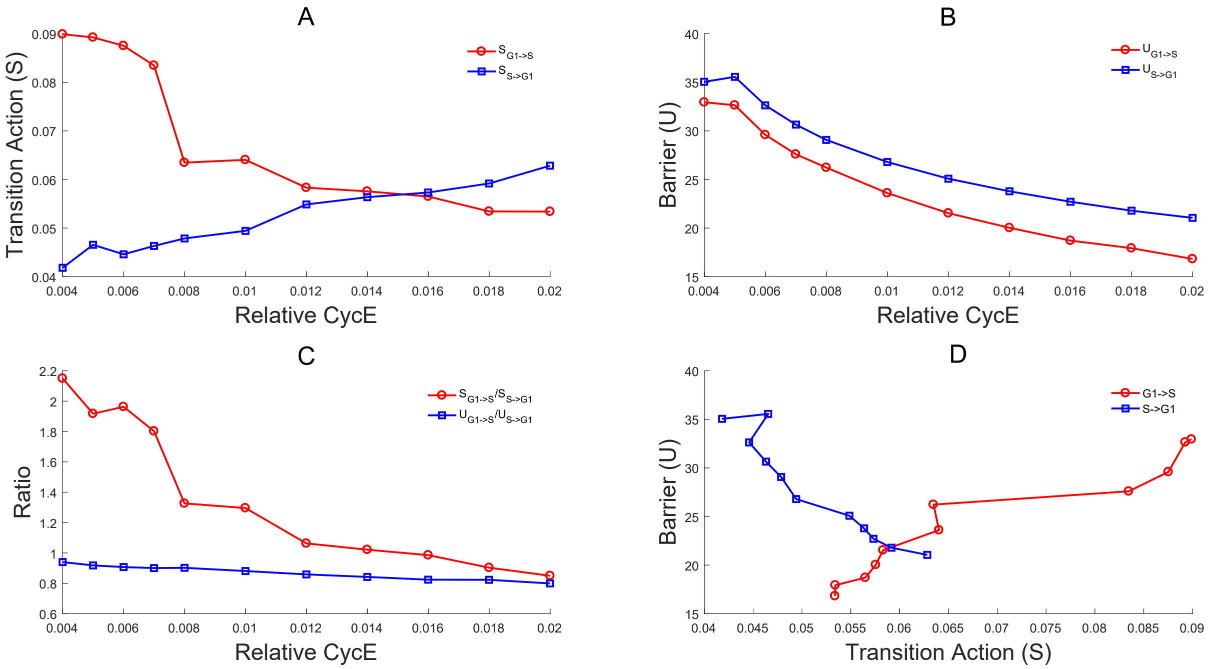


**Figure S14.** Effect of CycE (A-D) on the transition action (S) and barrier (U) between G1 and S. (A) Effect of CycE on the transition action between G1 and S. The x axis is the relative concentration of CycE, and the y axis is the value of transition action (S). The red line represents the G1->S action and the blue line represents the S->G1 action. (B) Effect of CycE on barrier (U) between G1 and S. The x axis is the relative concentration of CycE and the y axis is the barrier (U). The red line represents the G1->S barrier, and the blue line represents the S->G1 barrier. (C) The ratio of the action and the barrier between G1->S and S->G1 changes with CycE. The red line represents the ratio of the action between G1->S and S->G1. The blue line represents the ratio of the barrier between G1->S and S->G1. (D) Transition action (S) versus barrier (U). The red line represents the G1->S and the blue line represents S->G1.

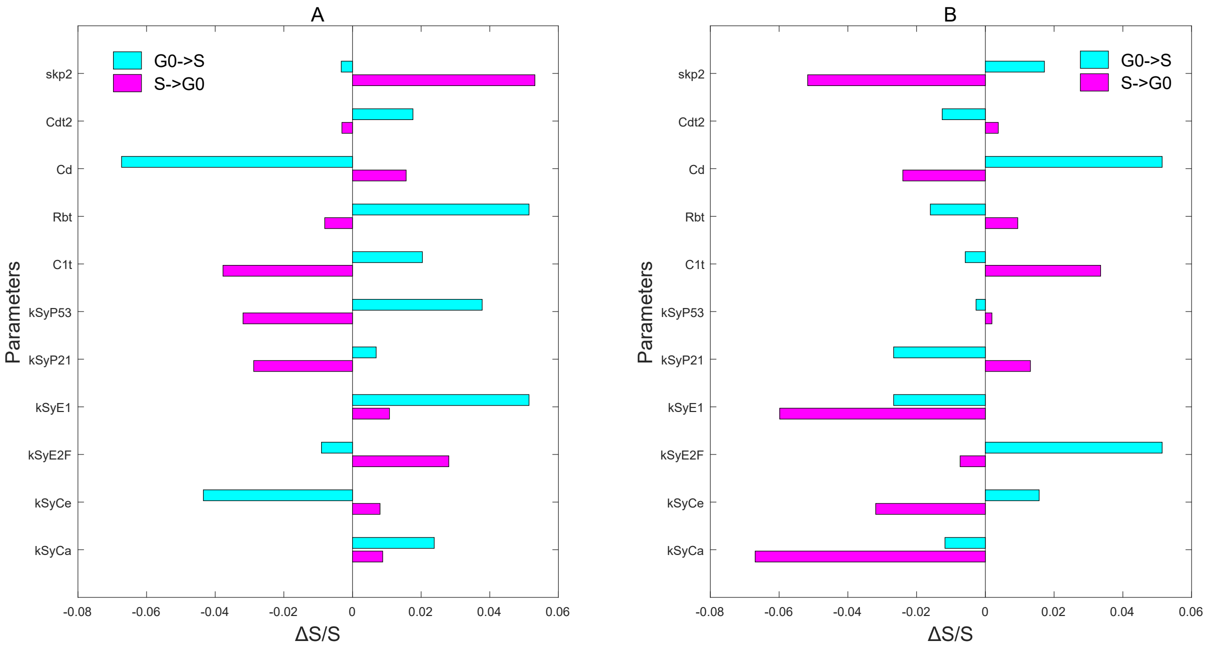


**Figure S15.** Sensitivity analysis for the 11 parameters, which control the synthesis of regulators, on the transition action of G0->S (A), S->G0 (B). The x axis represents the relative change of transition action in terms of unperturbed system, denoted by $\Delta S/S$. Some sensitive parameters, including kSyCe, kSyCa, kSyE2F, kSyP21, kSyP53, Rbt and Cd, are increased or reduced by 0.5%, while each of the other parameters is increased or reduced by 5%. Magenta bar represents the S->G0 transition, and cyan bar represents the G0->S transition. (A) Each parameter is increased for the transition between G0 and S. (B) Each parameter is decreased for the transition between G0 and S.

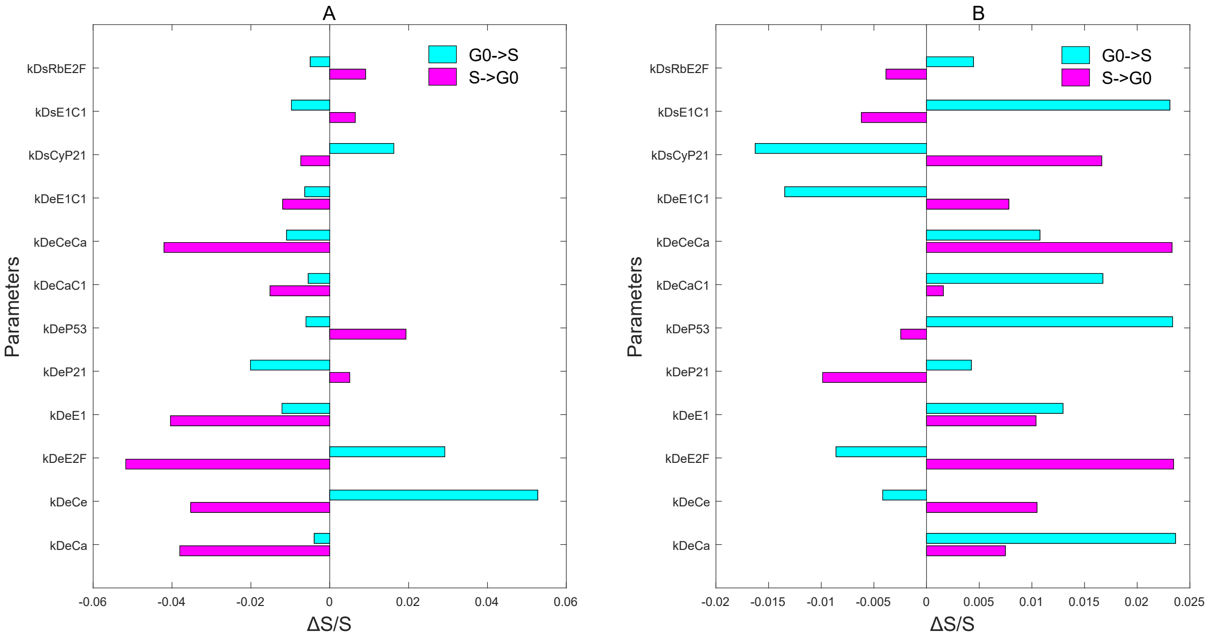


**Figure S16.** Sensitivity analysis for the 12 parameters, which control the degradation of regulators, on the transition action of G0->S (A), S->G0 (B). The x axis represents the relative change of transition action in terms of unperturbed system, denoted by $\Delta S/S$. Some sensitive parameters, including kDeCe, kDeE2F, kDeP21, kDeP53, are increased or reduced by 0.5%, while each of the other parameters is increased or reduced by 5%. Magenta bar represents the S->G0 transition, and cyan bar represents the G0->S transition. (A) Each parameter is increased for the transition between G0 and S. (B) Each parameter is decreased for the transition between G0 and S.

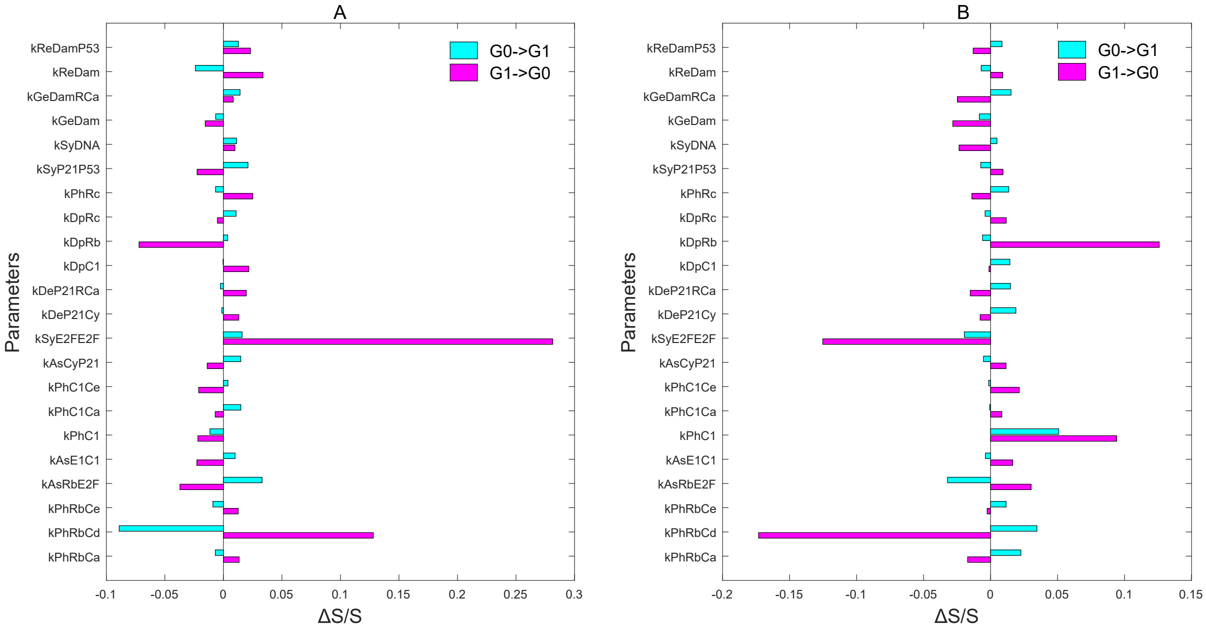


**Figure S17.** Sensitivity analysis for the 22 parameters, which control the interaction between regulators, on the transition action of G0->G1 (A), G1->G0 (B). The x axis represents the relative change of transition action in terms of unperturbed system, denoted by $\Delta S/S$. Some sensitive parameters, including kPhRbCd, kSyE2FE2F, kDpRb, kSyP21P53, kGeDam, are increased or reduced by 0.5%, while each of the other parameters is increased or reduced by 5%. Magenta bar represents the G1->G0 transition, and cyan bar represents the G0->G1 transition. (A) Each parameter is increased for the transition between G0 and G1. (B) Each parameter is decreased for the transition between G0 and G1.

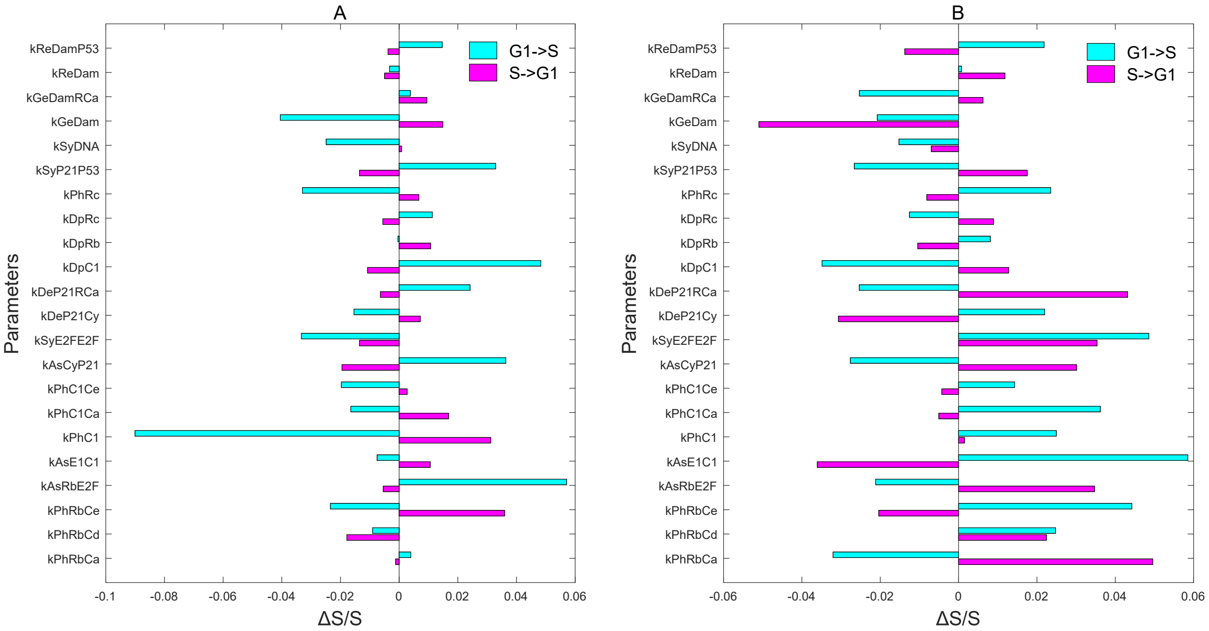


**Figure S18.** Sensitivity analysis for the 22 parameters, which control the interaction between regulators, on the transition action of G1->S (A), S->G1 (B). The x axis represents the relative change of transition action in terms of unperturbed system, denoted by ΔS/S. Some sensitive parameters, including kPhRbCd, kSyE2FE2F, kDpRb, kSyP21P53, kGeDam, are increased or reduced by 0.5%, while each of the other parameters is increased or reduced by 5%. Magenta bar represents the S->G1 transition, and cyan bar represents the G1->S transition. (A) Each parameter is increased for the transition between G1 and S. (B) Each parameter is decreased for the transition between G1 and S.

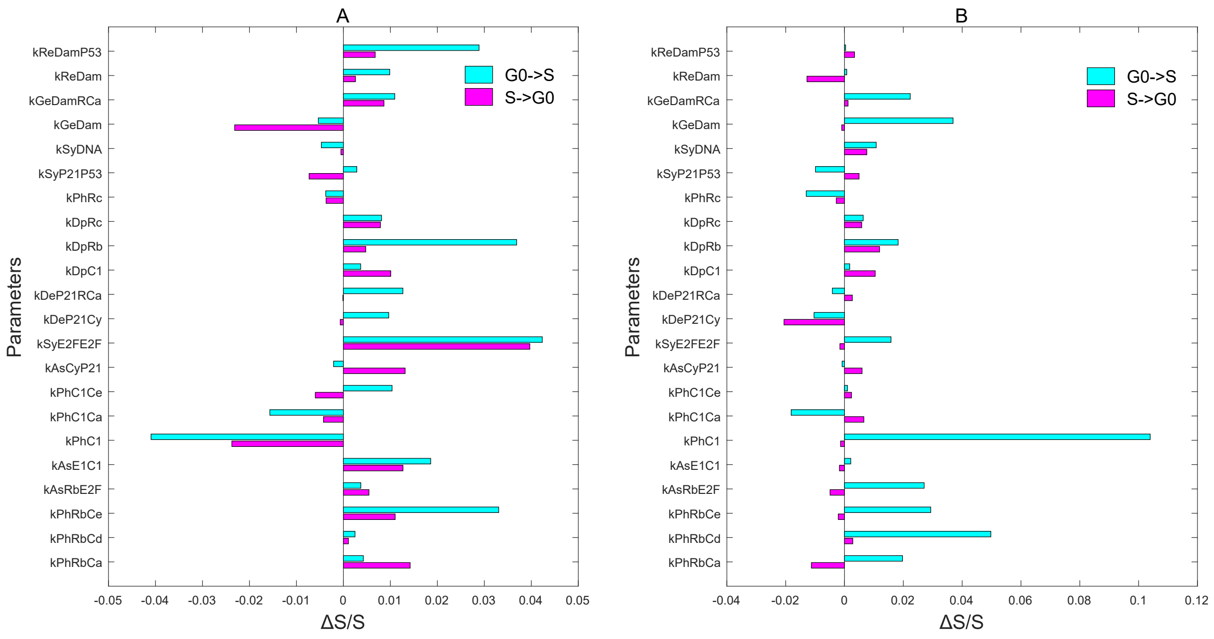


**Figure S19.** Sensitivity analysis for the 22 parameters, which control the interaction between regulators, on the transition action of G0->S (A), S->G0 (B). The x axis represents the relative change of transition action in terms of unperturbed system, denoted by ΔS/S. Some sensitive parameters, including kPhRbCd, kSyE2FE2F, kDpRb, kSyP21P53, kGeDam, are increased or reduced by 0.5%, while each of the other parameters is increased or reduced by 5%. Magenta bar represents the S->G0 transition, and cyan bar represents the G0->S transition. (A) Each parameter is increased for the transition between G0 and S. (B) Each parameter is decreased for the transition between G0 and S.

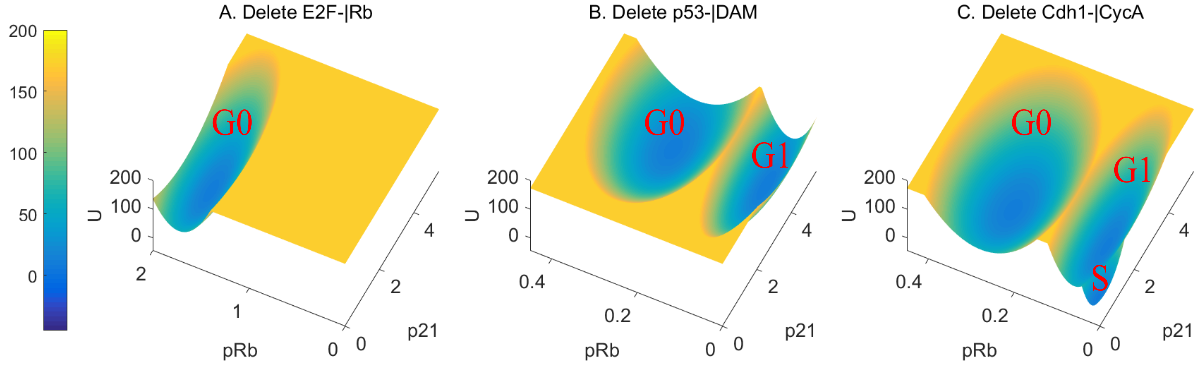


**Figure S20.** Random edge deletion causes landscape alteration. (A) Delete the inhibitory effect of E2F on Rb. (B) Delete the inhibitory effect of p53 on DNA damage. (C) Delete the inhibitory effect of Cdh1 on CycA. The U axis is the $-ln(P_{ss})$, $P_{ss}$ represents the steady states probability distribution.

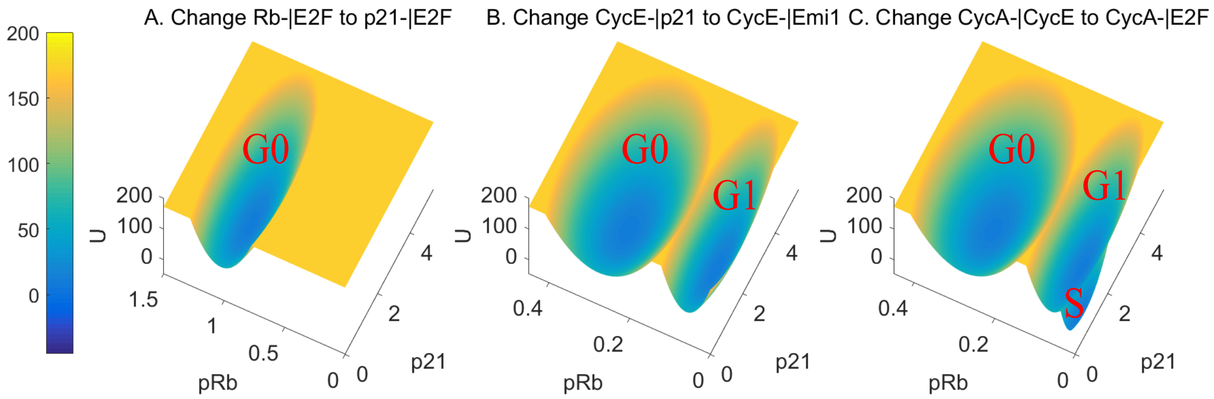


**Figure S21.** Random edge reassignment causes landscape alteration. (A) Change the inhibitory effect of Rb on E2F to the inhibitory effect of p21 on E2F. (B) Change the inhibitory effect of CycE on p21 to the inhibitory effect of CycE on Emi1. (C) Change the inhibitory effect of CycA on CycE to the inhibitory effect of CycA on E2F. The U axis is the $-ln(P_{ss})$, $P_{ss}$ represents the steady states probability distribution.

# References


1. Heldt FS, Barr AR, et al (2018) A comprehensive model for the proliferation–quiescence decision in response to endogenous dna damage in human cells. Proc Natl Acad Sci USA 115: 2532-2537.

2. Gookin S, Min M (2017) A map of protein dynamics during cell-cycle progression and cell-cycle exit. PLOS Biology 15: 1–25.

3. Tibshirani R, Walther G, et al (2002) Estimating the number of clusters in a data set via the gap statistic. Journal of the Royal Statistical Society: Series B 63: 411-423.

4. Dick F, Goodrich D, Sage J (2018) Non-canonical functions of the RB protein in cancer. Nature Reviews Cancer 18: 442–451.

5. Chung M, Liu C, Yang HW, et al (2019) Transient Hysteresis in CDK4/6 Activity Underlies Passage of the Restriction Point in G1. Molecular Cell 76: 1-12.

6. Furuno N, Pines EJ, et al (1999) Human Cyclin A Is Required for Mitosis until Mid Prophase. Journal of Cell Biology 147: 295-306.

7. Elzen N, Pines J (2001) Cyclin A Is Destroyed in Prometaphase and Can Delay Chromosome Alignment and Anaphase. Journal of Cell Biology 153: 121-135.

8. Frolov MV, Dyson NJ, et al (2004) Molecular mechanisms of E2F-dependent activation and pRB-mediated repression. Cell Sci 117: 2173-2181.

9. Yao G, Lee TJ, et al (2008) A bistable Rb-E2F switch underlies the restriction point. Nature Cell Biology 10: 476-482.
